# 3D HucMSCs derived extracellular vesicles enhanced therapeutic efficacy in treating intrauterine adhesions via BECN1 delivery

**DOI:** 10.1016/j.mtbio.2026.103276

**Published:** 2026-05-29

**Authors:** Juan Peng, Juan Zhang, Qingzhao Li, Weijun Liu, Wenda Zou, Liyu Zhu, Qianyin Zhou, Dan Liu, Man Jia, Hui Li

**Affiliations:** a Reproductive and Genetic Medicine Department, Zhuzhou Hospital Affiliated to Xiangya School of Medicine, Central South University, Zhuzhou, Hunan, China; b Translational Medicine Center, Zhuzhou Hospital Affiliated to Xiangya School of Medicine, Central South University, Zhuzhou, Hunan, China; c Hematology Department, Zhuzhou Hospital Affiliated to Xiangya School of Medicine, Central South University, Zhuzhou, Hunan, China

**Keywords:** Mesenchymal stem cells, Extracellular vesicles, Intrauterine adhesions, Fibrosis, 3D culture, BECN1

## Abstract

Intrauterine adhesion (IUA) is a significant cause of female infertility and a critical public health concern among women of childbearing age. Emerging evidence has highlighted the therapeutic potential of mesenchymal stem cell-derived extracellular vesicles (EVs) in IUA treatment. However, conventional two-dimensional (2D) culture systems impose constraints on the yield and biological activity of EVs. In this study, we employed a three-dimensional (3D) culture platform based on gelatin methacrylate (GelMA) microspheres to culture human umbilical cord mesenchymal stem cells and isolate MSC-EVs. Comparative analysis demonstrated that 3D-EVs exhibited significantly superior therapeutic effects on restoring the proliferation, migration, viability, and alleviating fibrosis of mifepristone-injured human endometrial stromal cells (hESCs) compared with 2D-EVs *in vitro*. In IUA animal models, *in situ* treatment with GelMA-encapsulated 3D-EVs exhibited remarkable regenerative capabilities. The treatment effectively rejuvenated endometrial structure including mucosal thickness and glandular density, ameliorated endometrial fibrosis, and ultimately improved reproductive outcomes. Results from proteomic profiling and functional verification assays revealed that elevated expression of BECN1 in 3D-EVs contributes to the enhanced therapeutic effect of 3D-EVs on injured hESCs. This study establishes 3D culture-derived EVs as an innovative cell-free therapeutic approach for endometrial regeneration, addressing both the limitations of traditional EVs production methods and the clinical need for effective IUA treatments. Our findings provide a foundation for advanced regenerative strategies targeting endometrial pathologies.

## Highlights

• Endometrial regeneration, scar remodeling, and fibrosis inhibition are the core therapeutic strategies for IUA treatment.
• MSC-EVs can promote endometrial regeneration, regulate endometrial function, and facilitate key processes associated with embryo implantation.
• 3D-EVs show enhanced therapeutic efficacy compared with 2D-EVs in IUA treatment.
• BECN1 in 3D-EVs alleviates fibrotic damage.

## Graphical abstract

**Figure:**
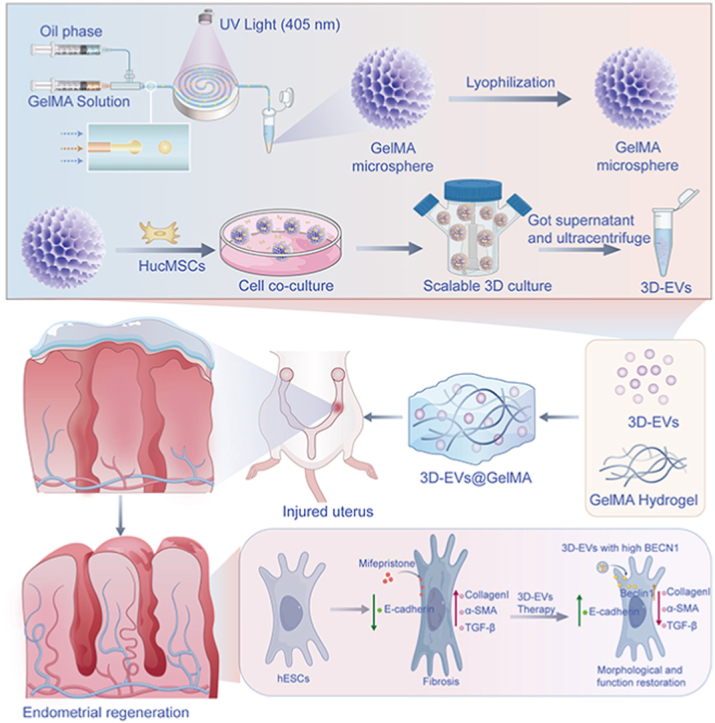

## 1 Introduction

Intrauterine adhesion (IUA) is the most common clinical manifestations of endometrial injury diseases. It is often caused by fibrous tissue hyperplasia and tissue adhesion after endometrial trauma, and the pathological process frequently leads to severe reproductive complications including amenorrhea, hypomenorrhea, infertility, recurrent pregnancy loss, and abnormal placentation [1,2]. The fundamental pathological mechanism underlying IUA involves impaired endometrial repair processes, particularly due to the depletion of functional stem cells in the basal layer of the endometrium. Promoting endometrial regeneration, facilitating scar remodeling, and suppressing fibrosis are keys points in therapeutic strategies [3]. Current clinical management of IUA involves a multimodal approach combining transcervical resection of adhesions (TCRA) with postoperative adjuvant therapies such as estrogen supplementation and intrauterine physical barriers [4]. However, these conventional treatments demonstrate significant limitations, including high recurrence rates, failure to restore endometrial regenerative capacity, and minimal improvement in reproductive outcomes [5]. Therefore, there is an urgent need to develop more effective therapeutic approaches to mitigate endometrial fibrosis, improve endometrial receptivity, avoid IUAs and recurrence, and ultimately improve clinical pregnancy outcomes.

Mesenchymal stem cell-derived extracellular vesicles (MSC-EVs) exhibit significant potential in promoting endometrial regeneration, reducing fibrosis and modulating endometrial function [[6], [7], [8]]. These nano-sized membrane vesicles (50-200 nm), which originate from endosomes, represent a promising cell-free therapeutic platform capable of transferring bioactive molecules including mRNA, miRNA, lipids, and proteins to modulate recipient cell functions. Compared to direct MSC transplantation, MSC-EVs offer several advantages: they maintain the stemness properties and regenerative capacity of their parent cells and address concerns related to oncogenic risk, immunogenicity, and low cellular retention rates [9,10]. Although EVs exhibit considerable therapeutic potential for IUA, their clinical application is hindered by several critical drawbacks, including low production yield, inadequate targeting efficiency, non-specific off-target accumulation, and rapid systemic clearance [11].

Conventional two-dimensional (2D) culture systems fail to sustain the stemness of MSCs, readily trigger cellular senescence, possess limited proliferative and expansive potential, and lack physiological microenvironmental cues. Consequently, EVs derived from such cultures exhibit low production yield and unstable biological functions [[12], [13], [14]]. The advent of three-dimensional (3D) culture systems has revolutionized cell culture methodologies by more accurately recapitulating the native tissue architecture and cellular complexity, while enhancing cell-cell and cell-matrix interactions. This advanced culture platform offers superior spatial efficiency and facilitates large-scale cell production [15]. Accumulating evidence has demonstrated that 3D culture significantly boosts EV yields and alters the cargo composition of EVs. Quantitative analysis revealed an 18.49-fold increase in RNA content and a 30-fold enhancement in EV production derived from microbead-based 3D-hMSCs compared to their 2D counterparts [16]. Furthermore, EVs derived from microfiber-based 3D-MSCs exhibited a 1009-fold enrichment in particle numbers alongside a broader proteomic profile (1023 vs. 605 identified proteins) relative to 2D controls [17]. The changes in the cargos of EVs are attributed to the cultural microenvironment. Distinct mechanical cues from the culture environment can trigger specific alterations in gene expression within MSCs while preserving their intrinsic properties. This, in turn, leads to changes in the cargos of EVs, such as cytokines, microRNAs, and proteins, etc [18]. As 3D culture provides a more physiologically relevant growth microenvironment, which preserves stem cell stemness and mitigates cellular senescence. Consequently, this leads to the improvement of both the quality and functional potency of secreted EVs [10]. Deng et al. validated that EVs from 3D-MSCs-EVs expressed higher levels of ANXA1 and exhibited superior *in vivo* wound healing promotion compared to 2D-MSCs-EVs [19]. 3D-adipose-derived stem cells (ADSCs)-EVs, cultivated via coaxial bioprinted microfibers, demonstrated enhanced therapeutic efficacy over 2D-ADSCs-EVs, as evidenced by improved renal function and alleviation of fibrosis in a mouse model of unilateral ischemia-reperfusion injury [20]. Additionally, 3D culture enhance the level of anti-inflammatory factor GAS6 in sEVs, and modulate microglia polarization after traumatic brain injury [21]. A phase I clinical trial further indicated that aerosolized 3D-hUCMSC-EVs, in combination with conventional therapy, significantly improved lung function indices and respiratory health without inducing serious adverse events, suggesting a promising novel strategy for pulmonary fibrosis treatment [22]. Cumulatively, studies across multiple disease models, including bone defects, cutaneous wound healing, liver injury, and renal injury, have consistently confirmed that 3D-EVs possess enhanced regenerative, anti-fibrotic, and anti-inflammatory efficacy compared to 2D-EVs [20,[23], [24], [25]].

Increasing 3D cell culture methods were developed to increase EVs efficiency. Although each of these methods has its own advantages, there are also disadvantages [26]. For instance, 3D coaxial bioprinting technology require expensive equipment and complex process. In addition, there is also the problem of easy cell contamination [20,25]. Bioreactor-based methods prone to causing excessive cell aggregation and significant shear stress-induced cell death [27]. Microcarrier method poses challenges in terms of cell loading and uniform distribution, and it also requires strict cultivation conditions [28,29]. Additionally, the conventional spheroid culture system relying on low-adhesion surfaces faces inherent challenges in maintaining uniform spheroid size, thereby inevitably inducing phenotypic and functional heterogeneity in the resultant extracellular vesicles [30]. Gelatin methacrylate (GelMA) microspheres (GMs) provide a biomimetic 3D microenvironment that more closely resembles the native extracellular matrix, which better maintains the stemness and paracrine activity of MSCs compared with conventional scaffold-free 3D spheroids [31,32]. Benefiting from its high surface area-to-volume ratio, uniform morphology, and biomimetic characteristics, this system significantly enhances cell culture efficiency and establishes highly standardized, reproducible culture conditions [33]. Compared to other 3D culture systems, GMs culture system is simpler, more controllable, and more reproducible for laboratory-scale EV preparation, while still achieving superior EV yield and therapeutic efficacy [32]. ADSCs cultured in GMs display uniform spatial distribution and demonstrate significantly enhanced cell viability, attachment, proliferation, metabolic activity, and differentiation potential relative to 2D culture conditions [34]. Similarly, human dental pulp stem cells encapsulated within GMs show improved survival rates and superior extracellular matrix protein production compared to their 2D-cultured counterparts, highlighting their remarkable potential as cell delivery vehicles for endodontic regeneration applications [35]. Their physicochemical properties position GMs as an ideal biomaterial platform for diverse applications, including cell-based therapies, tissue engineering approaches, and 3D-EVs production.

A major drawback of EVs administration is their rapid systemic clearance *in vivo*. Furthermore, the off-target effects of EVs impede their efficient accumulation at lesion sites and may instead lead to undesirable deposition in non-target tissues [11]. Therefore, the development of delivery systems that enable controlled, sustained release and *in situ* delivery of EVs for endometrial repair is critical for the clinical translation of EV-based therapies in IUA treatment. GelMA hydrogel is a widely utilized injectable biomaterial in tissue engineering, owing to its outstanding biocompatibility, biodegradability, and capacity to encapsulate diverse bioactive molecules [36,37]. This biomaterial closely mimics the native extracellular matrix (ECM) microenvironment and incorporates the arginine-glycine-aspartic acid (RGD) tripeptide motif, which exerts critical regulatory effects on cellular behaviors such as adhesion, migration, and multi-lineage differentiation. Meanwhile, GelMA contains enzymatically degradable sequences that are sensitive to matrix metalloproteinases (MMPs); these sequences are essential for wound healing, tissue morphogenesis, and regenerative processes [38]. Furthermore, its photo-crosslinkable property enables facile *in situ* gelation upon injection [39]. Growing evidence demonstrates that GelMA hydrogels can serve as effective carriers for localized EVs delivery, which significantly enhances the bioavailability and sustained release of EVs *in vivo* [40,41].

The pathological manifestation of IUA is endometrial fibrosis; however, its underlying pathogenesis remains incompletely understood. Autophagy, an evolutionarily conserved intracellular pathway responsible for degradation and recycling, plays a pivotal role in maintaining cellular homeostasis, orchestrating stress responses, and regulating developmental processes [42]. Accumulating evidence indicates that autophagy activity *in vivo* is closely linked to the development of organ fibrosis [43]. For instance, inhibition of autophagy in hepatic stellate cells has been shown to ameliorate hepatic fibrosis [44]. Conversely, enhancement of autophagy via BECN1 (Beclin1) improved renal outcomes and reduced fibrosis in Unilateral Ureteral Obstruction mice [45]. Similarly, autophagy exerts a critical function in the progression of endometrial fibrosis [46]. Previous studies have confirmed that autophagy level in the endometrium of IUA patients are significantly reduced, and this inhibition further promotes fibrosis by modulating the EMT process [47]. Long et al. demonstrated that in SMAD7 conditional knockout mice, the endometrium displayed a fibrotic phenotype accompanied by impaired autophagic flux. Their findings further established that abrogation of SMAD7 drives endometrial fibrosis through the suppression of autophagic flux [48]. Collectively, these studies suggest that autophagic flux may represent a potential therapeutic target for the intervention of IUA. Notably, mechanical stress has been reported to modulate both autophagy initiation and autophagosome maturation [49]. Hydrogels, as ideal 3D cell culture scaffolds, providing a structural basis for mechanical stimulus-induced autophagy [50]. For instance, moderate static compression (2.5 g/cm^2^) imposed on human periodontal ligament cells seeded in hydrogels markedly upregulates BECN1 and LC3 expression via the AKT-mTOR signaling pathway, verifying that compressive mechanical force of 3D culture system drives autophagy activation [51,52]. Consequently, such autophagy alterations further reshape the autophagy-associated cargo profiles of secreted EVs.

In this study, we isolated 3D-EVs from human umbilical cord mesenchymal stem cells (HucMSCs) using a GMs culture system and investigated their therapeutic potential for IUA. First, an injured human endometrial cell (hESC) model was established to validate the restorative effects of 3D-EVs *in vitro*. Subsequently, we incorporated 3D-EVs into GelMA hydrogels to evaluate their therapeutic efficacy in a IUA mouse model. Finally, transcriptomic and proteomic sequencing were conducted to explore the potential mechanism. We ultimately identified that the autophagy-related protein BECN1, which was more highly expressed in 3D-EVs, plays a key role in mediating the therapeutic effects against IUA. This study provides novel insights into IUA treatment and lays the foundation for translating 3D-EVs into clinical practice.

## 2 Materials and methods

### 2.1 Isolation and characterization of HucMSCs

This study was approved by the Human Research Ethics Committee of Central South University Zhuzhou Hospital (Approval No.: KY2025109-01). Umbilical cord tissues were collected from three healthy parturients undergoing cesarean section (n = 3, aged 20-25 years, gestational age: 37-42 weeks) at Central South University Zhuzhou Hospital (Zhuzhou, China) and immediately placed in PBS following delivery. All donors were screened for infectious diseases, including human immunodeficiency virus (HIV), hepatitis B virus (HBV), hepatitis C virus (HCV), and syphilis, with all results confirmed negative. Donors with pregnancy complications, relevant medical histories, or other risk factors that might compromise the quality of umbilical cord–derived cells were excluded. Prior to sample collection, written informed consent was obtained from all donors in accordance with the ethical guidelines. The Wharton's jelly (WJ) region of umbilical cords was cut into 2 mm fragments, followed by digestion with collagenase II (Sigma, USA) and trypsin (Gibco, USA). The digested tissues were then filtered through a 100 μm mesh, and the resulting cell suspension was cultured in mesenchymal stem cell culture medium at 5% CO_2_ and 37 °C. After three days, the solution began to change every two days. When cells covered two-thirds of the dish surface, cell digestion and passage were performed. Each umbilical cord sample was processed and expanded independently to establish a unique HucMSC line. The 2D/3D-EVs used in the same experiment were originated from the same HucMSC line. The morphology of the HucMSCs was evaluated using an inverted fluorescence microscope. The expression of surface markers CD73, CD90, CD105, and CD45 in the HucMSCs was detected through flow cytometry. All antibodies were procured from BD Biosciences. The multidirectional differentiation potential of HucMSCs was evaluated through experiments involving adipogenesis, osteogenesis, and chondrogenesis as previously described [53].

### 2.2 Extraction and identification of 2D-EVs and 3D-EVs

GMs (EngForLife, China) were used for 3D HucMSC culture. They were added into 96-well plates with 200 microspheres per well. Then, MSC chemical-defined culture medium (NC0103, Yocon, China) was added and allowed to swell completely at 37 °C for 2 h. Next, 200 μL of cell suspension containing 10,000 HucMSCs were introduced into each well. The plates were subsequently placed on a rocking platform inside an incubator, set at an 8° angle with a 20 rpm rotation speed. During the initial 24 h culture period, the HucMSCs progressively attached to the surface and expanded within the GMs. Then, all HucMSC@GMs were transferred to a bioreactor with a 20 rpm rotation speed. For harvesting supernatant, the medium was replaced every 24 h and replenished with an equal volume of fresh MSC chemical-defined medium. This culture and supernatant collection process continued uninterrupted for 7 days. For 2D culture, HucMSCs were planar cultured, and the cellular supernatant was collected 48 h later. 2D-EVs and 3D-EVs were isolated from the cellular supernatant by differentiation ultracentrifugation. EVs derived from 2D and 3D cultures (designated as 2D-EVs and 3D-EVs, respectively) were isolated from cell-conditioned supernatants via differential ultracententrifugation. Briefly, cell debris and non-viable cells were first removed by sequential centrifugation at 300*g* for 10 min, 2000 g for 20 min, and 10,000 g for 30 min. The clarified supernatant was then filtered through a 0.22 μm membrane to exclude larger particles. Subsequently, EVs were pelleted via two rounds of ultracentrifugation using a tabletop ultracentrifuge (Himac CP100WX, Hitachi) at 100,000 g for 90 min: the first centrifugation was performed with a P27A rotor, while the second spin utilized a P55ST2 rotor to resuspend the EVs in 1 mL of phosphate-buffered saline (PBS). All ultracentrifugation steps were carried out at 4 °C. The obtained EV pellets were either immediately resuspended in PBS for subsequent downstream experiments or cryopreserved at −80 °C for long-term storage. All functional assays were completed within 14 days post-isolation to ensure vesicle integrity.

The morphology and size of distribution of EVs were observed by transmission electron microscopy (TEM; JEOL, Japan) and nanoparticle tracking analysis (NTA; Particle Metrix, Germany). Western blotting was employed to detect EVs markers [CD81, TSG101, CD63, and calnexin (Abcam, USA)].

### 2.3 Preparation of EVs-loaded GelMA hydrogels

GelMA was purchased from EFL (Eng For Life, China), mixed with 0.5% photoinitiator lithium acylphosphinate solution at a 1:5 mass ratio, stirred while heating to 60 °C to prepare 20% GelMA solution, then cooled to 37 °C. The concentrated EVs solution (2 × 10^9^ particles/mL) was combined with an equal volume of 20% GelMA solution and incubated at 37 °C on a shaker for 30 min to obtain a precursor solution with a 10% concentration of GelMA loaded with EVs. The mixture was injected into a mold (with a diameter of 20 mm and a thickness of 20 mm) and allowed to cure and shape under 405 nm UV light for 30 s to form EVs@GelMA hydrogels.

### 2.4 Cell culture and treatment

hESCs were purchased from Shanghai Zhong Qiao Xin Zhou Biotechnology Company (#ZQ-Y064, ZQXZ-bio, China) and cultured in hESC complete culture medium (PCM-H-069, ZQXZ-bio, China) at 5% CO_2_ and 37 °C. When the cell fusion rate was approximately 80%, the cells were cultured with medium containing 60 μmol/L mifepristone (Mif) (Sigma, USA) for 48 h. After that, hESCs were incubated with fresh culture medium or fresh culture medium containing 2D-EVs or 3D-EVs (at concentrations of 5 × 10^8^ particles/mL, 1 × 10^9^ particles/mL, 5 × 10^9^ particles/mL, or 1 × 10^10^ particles/mL) for an additional 2, 4, or 6 days. hESCs cultured without Mif and EVs were used as negative control (NC).

### 2.5 Construction of endometrial injury mice models and therapeutic treatment

The animal care and experiments were approved by Institutional Animal Care and Use Committee of Central South University (XMSB-2025-0370). A total of 100C57/BL6 Female mice (aged 8-10 weeks; 22-25 g) were utilized. The mice were housed in the hospital's animal facility. The animals were randomly allocated to five groups (Sham, IUA, Gel, 2D-EVs@Gel, 3D-EVs@Gel) through a lottery box method. Another assistant, who was unaware of the group assignments, carried out the IUA procedure. Sham group: abdomen exposure without uterus injury or hydrogel implantation; IUA group: uterus injury followed by 100 μL of PBS injection at injury site; Gel group: uterus injury with implantation of 100 μL of GelMA hydrogel on injury site; 2D-EVs@Gel group: uterus injury with implantation of 100 μL of GelMA hydrogel loaded with 2D-EVs (1 × 10^10^ particles) at injury site; 3D-EVs@Gel group: uterus injury with implantation of 100 μL of GelMA hydrogel loaded with 3D-EVs (1 × 10^10^ particles) at injury site. IUA model was established by mechanical injury. Briefly, anesthesia was induced with 5% isoflurane and maintained at 1.5% isoflurane. The mice's abdominal hair was then removed and the skin disinfected. A longitudinal 2 cm incision was made on the lower abdomen. The Y-shaped mouse uterus was found, and the uterine cavity was completely scraped with a micro endometrial scraper. The scraping was stopped when obvious congestion was observed in the uterine body and the uterine wall became rough. After clamping, 80% anhydrous ethanol was infused for 30 s, followed by rinsing with normal saline, and then the skin was sutured. After 7 days, reopen the ababdomen, the PBS, GelMA, 2D-EVs@Gel, or 3D-EVs@Gel was implanted at the injury site. For histological analysis, after 14 days, a subset of mice (n = 7 per group) was sacrificed. The harvested uteruses were subsequently fixed in PFA for 24 h at 4 °C before being processed for paraffin embedding. Another subset of mice (n = 5 per group) was sacrificed and their uteruses were collected and promptly frozen in liquid nitrogen for Western blot analysis.

### 2.6 Sustained release of DiR-labeled EVs from GelMA hydrogel *in vivo*

EVs were labeled with DiR (CSA:10006, MCE) by incubating at 37 °C for 30 min. Unlabeled DiR was subsequently removed via ultracentrifugation (100,000 × g, 120 min), then DiR-EVs were mixed with GelMA solution to form EVs@GelMA hydrogel. The DiR-EVs@GelMA was transplanted into the uteruses of mice. Photographs were taken and analyzed using an *in vivo* imaging system (AniView100, China).

### 2.7 Fertility and live birth assessment

Three estrous cycles after EVs-GelMA administration, female mice were paired with healthy 8-10-week-old male mice at a 2:1 ratio for a 96-h cohabitation period. Vaginal plugs were checked daily; once a vaginal plug was detected, a vaginal smear was prepared to observe the presence of sperm. The day sperm were observed in the vaginal smear was defined as gestational day 0.5. Females with no detectable sperm were excluded from subsequent analyses during the cohabitation period. Embryo implantation was examined 14 days after mating, and changes in maternal body weight were recorded. Some female mice were allowed to deliver naturally, and the number of fetuses, number of live births, and fetal weight on postnatal day 1 and day 14 were documented.

### 2.8 Transcriptome sequencing

hESCs were exposed to Mif for 48 h to induce injury, followed by treatment with either PBS, 2D-EVs, or 3D-EVs for 48 h, and then subjected to transcriptome sequencing. Each group contained four biological replicates. hESCs without Mif and EVs treatment were used as NC. In brief, total RNA was isolated from the hESCs with RNAex Pro reagent (Accurate Biotechnology, China). RNA integrity was verified prior to library preparation. Sequencing libraries were constructed and processed on the PromethION platform (Biomarker Technologies, China). Raw sequencing data quality was evaluated using FastQC and MultiQC, followed by adapter trimming and quality filtering with Trimmomatic. Gene-level quantification was performed by mapping reads to the reference genome. Transcriptomic variations across experimental groups were visualized through principal component analysis (PCA) implemented in the factoextra package. For fibrosis-related gene discovery, expression patterns were clustered using Mfuzz with a membership threshold >0.5. Functional annotation of cluster-specific genes was conducted via GO biological processes (GO-BP) and KEGG pathway enrichment analyses using clusterProfiler.

### 2.9 Proteomic analysis

2D-EVs and 3D-EVs from three biological replicates were lysed and then subjected to nano-UHPLC-MS/MS analysis (TimS TOF Pro 2, Bruker). Intensity-based absolute quantification was used for label-free quantification in Scaffold Viewer v5.0 (Proteome Software), with the criteria of at least 2 unique peptides per protein and a false discovery rate below 1% at both the protein and peptide levels. Only proteins detected consistently across all replicates within each group were included for comparative analysis. EVs protein classification was performed against Vesiclepedia 5.1 using the ggvenn R package, while differential expression analysis employed the limma package with significance thresholds of adjusted *p* < 0.05 (Benjamini-Hochberg method) and |log2(FC)| > 0.585. Functional annotation and enrichment analysis were conducted using Metascape (http://metascape.org).

### 2.10 BECN1 shRNA lentivirus transfection

For the knockdown of BECN1 within 3D-EVs, shRNA sequences (shBeclin1-1, shBeclin1-2, shBeclin1-3) (Table S2) targeting the BECN1 gene and a negative control shRNA (shNC) were designed and chemically synthesized by Hanbio Technology (Shanghai, China). HucMSCs were transduced with shBECN1 lentivirus at a multiplicity of infection (MOI) of 40 in accordance with the operational protocols of the manufacturer. Then HucMSCs were expanded in 15 cm culture plates to a total cell quantity of 3 × 10^8^. Afterward, these cells were subjected to 3D culture with GMs to generate 3D-EVs. Cell culture supernatants were harvested at 24-h intervals.

### 2.11 Other methods

Other methodologies were presented in the supplementary material, including fluorescence labeling of EVs, wound healing and cell migration assays, cell viability and live/dead assays, cell fibrosis assay, *in vitro* release assay of EVs from hydrogel, hematoxylin eosin (HE), RT-qPCR, Masson and immunohistochemical (IHC) staining.

### 2.12 Statistical analysis

Results are expressed as means with their standard deviations, and statistical tests were carried out using Graph Pad Prism 9.0. Student's *t*-test analyzed significance between two groups, while one-way ANOVA assessed significance among several groups. When significant differences were detected in the ANOVA, Bonferroni or Tukey post hoc tests were performed for pairwise comparisons. In figures, ∗ stands for *p* < 0.05, ∗∗ for *p* < 0.01, and ∗∗∗ for *p* < 0.001; ns stands for no significance.

## 3 Results

### 3.1 Isolation and identification of HucMSCs

HucMSCs were isolated from the umbilical cords of newborns. Fig. S1 shows the isolation and culture protocol for HucMSCs. After 20-30 days of primary culture, cells displayed fibroblast-like spindle-shaped morphology (Fig. S1C). Flow cytometric analysis was used to confirm the immunophenotypic profile of HucMSCs. Results suggested high expression of canonical MSC markers: CD73 (96.2%), CD90 (97.3%), and CD105 (98.1%). Concurrently, minimal expression of the hematopoietic marker CD45 (1.81%) was observed (Fig. S1B). These findings illustrated that the isolated HucMSCs had high purity and mesenchymal identity.

Additionally, the trilineage differentiation capacity of HucMSCs was validated through standardized induction protocols (Fig. S1C). Under osteogenic conditions, cells formed mineralized nodules visualized via Alizarin Red staining, confirming calcium deposition and osteoblast differentiation. Adipogenic induction resulted in lipid vacuole accumulation, evidenced by Oil Red O staining, indicative of mature adipocyte formation. Chondrogenic differentiation was demonstrated through pellet culture systems, where Alcian Blue staining revealed glycosaminoglycan deposition, characteristic of cartilage matrix formation. These functional assays collectively validated the multipotent nature of HucMSCs, a hallmark feature of MSCs with implications for tissue regeneration and therapeutic applications.

### 3.2 Isolation and characterization of 2D-EVs and 3D-EVs

The process for 3D-EVs preparation and EVs collection is shown in Fig. 1A. The 3D culture of HucMSCs was achieved using GMs. Fig. 1B shows GMs exhibiting uniform spherical morphology, with HucMSCs demonstrating robust adherence and spreading across the microcarrier surface. Live/dead staining confirmed high viability of MSCs cultured on GMs for 7 days, validating the biocompatibility of this 3D culture system. These findings support the utility of GMs as microcarrier for scalable MSC expansion while maintaining cellular integrity. Afterward, we collected supernatant of 3D cultured HucMSCs and obtained EVs through ultracentrifugation. EVs were then identified by TEM, NTA, and Western blotting analysis. TEM results revealed that the isolated EVs had characteristic cup-shaped morphology in both 2D and 3D culture systems (Fig. 1C). According to NTA characterization, 2D-EVs and 3D-EVs showed similar size distributions, while 3D-EVs possessed a marginally smaller average particle diameter of 127.6 ± 63.3 nm in contrast with 2D-EVs, which presented a mean size of 135.2 ± 55.8 nm (Fig. 1D). Western blot analysis confirmed enrichment of canonical EVs markers (CD81, TSG101, and CD63) in both 2D-EVs and 3D-EVs, with absent calnexin expression (Fig. 1E). The above results indicated that we extracted high purity 2D-EVs and 3D-EVs. Comparative analysis (Fig. 1F) demonstrated that 3D culture systems produced approximately twice the EV particle concentration and 50% higher protein content per cell relative to conventional 2D cultures, suggesting 3D systems can optimize EVs production efficiency while maintaining protein cargo fidelity.

**Fig. 1:**
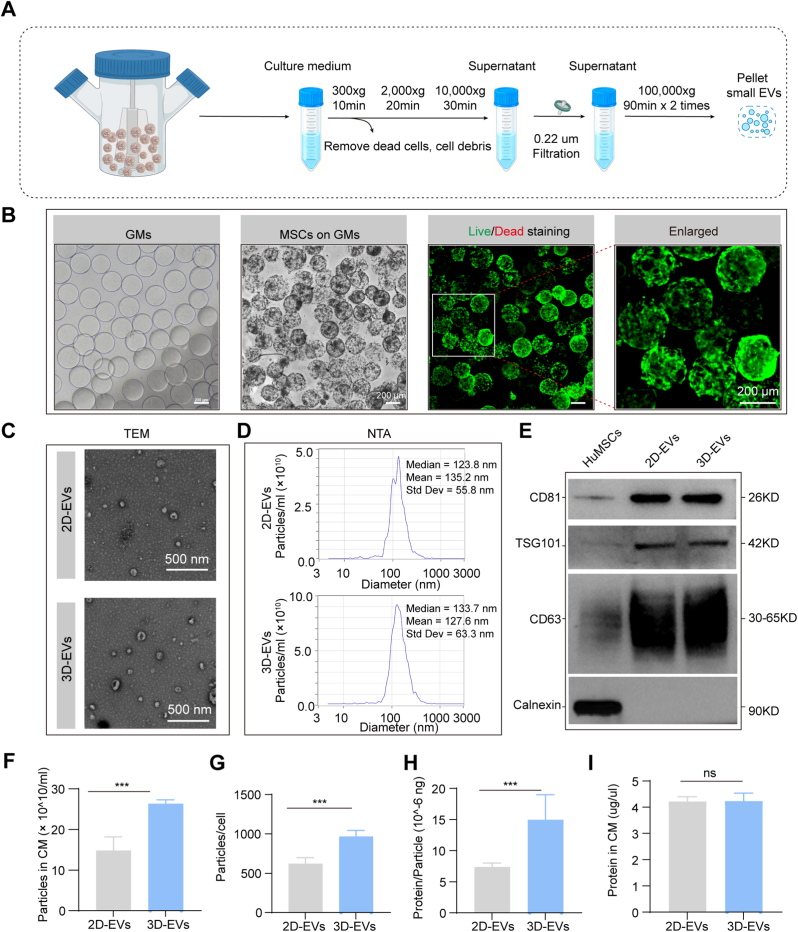
**Isolation and characterization of EVs derived from 2D/3D cultured HucMSCs.** A. The workflow for EVs production and isolation. B. The appearance of GelMA microspheres (GMs) with/without MSC adhesion (Left) and live/dead (green/red fluorescence) staining assay for MSCs (Right). C. Morphological of 2D-EVs and 3D-EVs evaluated by TEM. D. NTA analysis of the size distribution of 2D-EVs and 3D-EVs. E. Western blot analysis of the enrichment of EV markers (CD81, TSG101, CD63) in both 2D/3D-EVs. F. Quantitative analysis of the particle concentration and protein content of 2D/3D-EVs. Data are presented as mean ± SD, n = 3 independent biological replicates. CM: culture supernatant. (For interpretation of the references to colour in this figure legend, the reader is referred to the Web version of this article.)

### 3.3 3D-EVs protect against Mif-induced injury of hESCs

The endometrium has mesenchymal and epithelial components, with endometrial stromal cells serving as key regulators in the cyclic regeneration and remodeling of endometrial tissue [54]. To investigated the therapeutic potential of 3D-EVs compared to 2D-EVs in endometrium injury repair, we established an injured hESC model with Mif treatment. Fig. 2A outlines the experimental design. First, we used CCK-8 assay, RT-qPCR, and Western blot analysis to screen for the optimal dose (60 μM) and treatment duration (48 h) of Mif for establishing an hESC injury model (Figs. S2 and S3). Then we optimized the treatment dosage and duration for both EVs types. hESCs were initially exposed to Mif 48 h before receiving either 2D-EVs or 3D-EVs treatment. Cell viability was assessed using the CCK-8 assay and live/dead staining. As shown in Fig. 2B, CCK-8 results demonstrated a concentration-dependent enhancement of cell viability following treatment with either EVs type. The optimal therapeutic effect was achieved at a concentration of 1 × 10^9^ particles/mL for both 2D-EVs and 3D-EVs. Longitudinal monitoring of cell viability (Fig. 2D) demonstrated that while both EVs treatments improved cell survival over a 6-day period, 3D-EVs exhibited significantly greater and more sustained therapeutic effects from day 4 onward (*p* < 0.05). Then live/dead staining was used to examine cell viability after 4 days of treatment with 1 × 10^9^ particles/mL EVs. Both EVs-treated groups showed increased populations of viable cells (green fluorescence) compared to Mif-only controls, with 3D-EVs demonstrating markedly superior protective effects as evidenced by higher ratios of live cells (Fig. 2E and F).

**Fig. 2:**
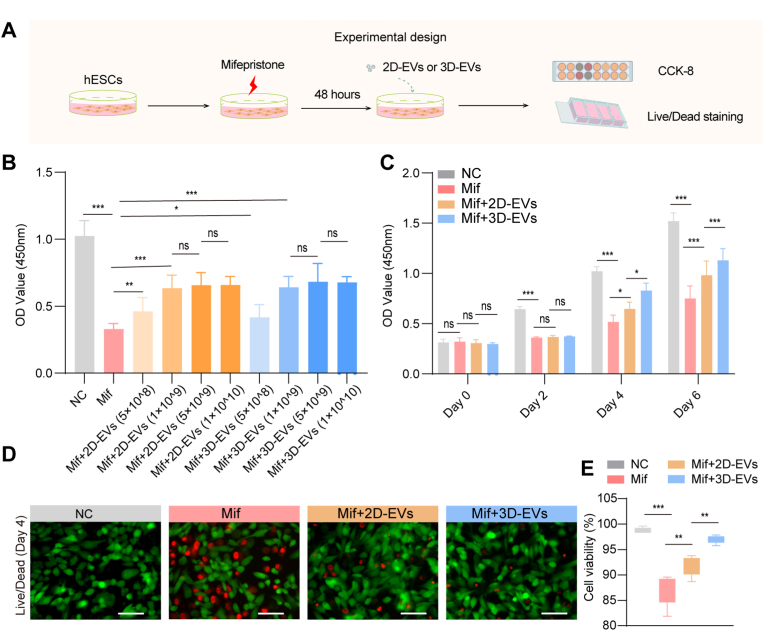
**Optimal dosage and timing exploration for EVs treatment of hESCs.** A. Experimental design. B. The viability of Mif-injured hESCs in response to different concentrations of 2D/3D-EVs was evaluated using the CCK-8 assay. C. Sustained viability of Mif-injured hESCs following treatment with 5 × 10^9^ 2D/3D-EVs. D. Fluorescent microscopy image of Live/dead assay for injury hESCs 4 days after 2D/3D-EVs treatment. Green means live and red means dead cells. E. Quantitative data of cell viability corresponding to D. ns, not significant versus the indicated group. The data represent means ± SD, n = 4 independent biological replicates. Mif: mifepristone. (For interpretation of the references to colour in this figure legend, the reader is referred to the Web version of this article.)

To evaluate the internalization dynamics of EVs, PKH26-labeled 2D-EVs and 3D-EVs were incubated with hESCs for 24 h. Confocal microscopy revealed co-localization of red fluorescence signals (PKH-26) with F-actin (green, cytoskeletal marker) and DAPI (blue, nuclear staining), confirming successful EVs internalization within cytoplasmic compartments. Subsequent functional assays compared the therapeutic effects of 3D-EVs versus 2D-EVs on injured hESCs. Transwell migration assays demonstrated that both EVs types significantly attenuated Mif-induced inhibition of cell migration (Fig. 3B and C). The 3D-EVs-treated group exhibited significantly more migrated cells compared to the 2D-EVs-treated group (*p* < 0.01). Wound healing assays corroborated these findings, both EVs treated groups showing significantly enhanced lateral migratory distance compared to the Mif-only group at 48 h post-scratch, with 3D-EVs-treated group significant outperformed 2D-EVs-treated group (Fig. 3D and E). These results highlight the enhanced effectiveness of 3D-EVs in promoting cell migration, which is a critical mechanism in tissue regeneration.

**Fig. 3:**
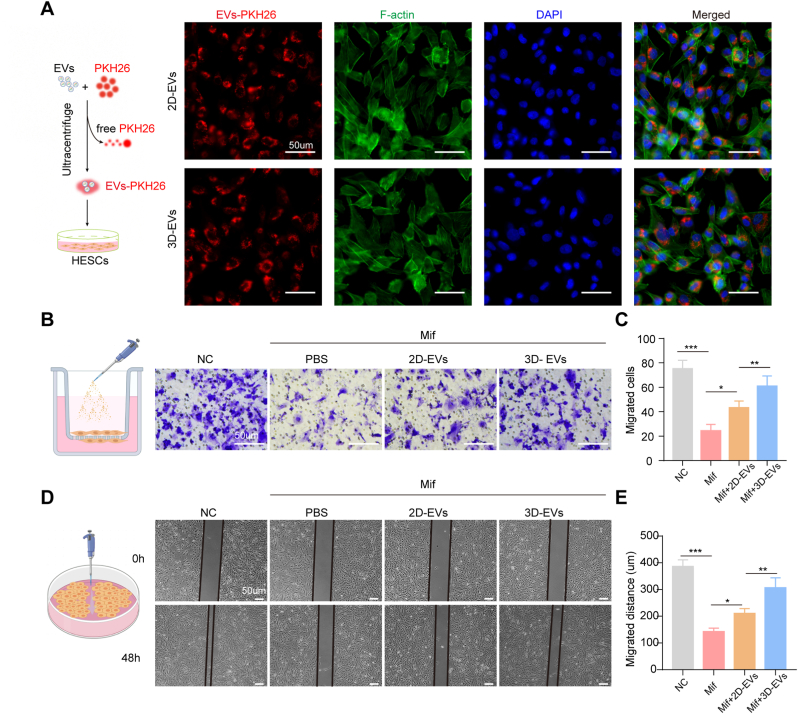
**3D-EVs protect against mifepristone-induced inhibition of HESCs migration.** A. Uptake of PKH-26-labeled EVs by hESCs. EVs showed red, F-actin showed green, and nuclei showed blue. B, C. Transwell assay images and quantitative analysis of migrated hESCs. D, E. Wound healing assay images and quantitative analysis for the migration distance of hESCs. The data represent means ± SD, n = 5 independent biological replicates. (For interpretation of the references to colour in this figure legend, the reader is referred to the Web version of this article.)

EdU proliferation assays revealed that Mif treatment significantly suppressed hESC proliferation compared to untreated controls (*p* < 0.001). Following 48 h incubation with either EVs subtype, proliferation rates were significantly restored (*p* < 0.01 vs. Mif group), with 3D-EVs demonstrating more pronounced pro-proliferative effects (Fig. 4A and B). In addition, the apoptosis ratio of cells was assessed by flow cytometry. As illustrated in Fig. 4C and D, Mif treatment significantly increased the proportion of apoptotic hESCs. Both 2D-EVs and 3D-EVs treatments effectively attenuated this apoptosis, with the 3D-EVs group demonstrating significantly lower apoptotic rates compared to 2D-EVs (*p* < 0.05). These findings suggest that EVs can alleviate Mif-induced apoptosis, with 3D-EVs exhibiting superior anti-apoptotic efficacy. To investigate the influence on fibrosis, the expression of collagen I in hESCs was examined via immunofluorescence. Fig. 4E and F shows significantly elevated collagen I accumulation in Mif-treated groups versus controls (*p* < 0.001). Subsequent EVs administration reduced fibrotic deposition, with 3D-EVs achieving a greater reduction than 2D-EVs (*p* < 0.05). Furthermore, Western blot analysis was conducted to assess the changes in fibrosis-related markers, including E-cadherin (E-cad), COL1A1, TGF-β, and α-SMA. The results reveal that Mif treatment led to a decrease in E-cad expression and an increase in the expression of COL1A1, TGF-β, and α-SMA compared to the NC group. However, both 2D-EVs and 3D-EVs reversed these Mif-induced changes, with 3D-EVs demonstrating a more significant regulatory effect on the expression of these fibrosis-related markers compared to 2D-EVs (Fig. 4G and H). These demonstrate that both EVs subtypes mitigate Mif-induced fibrosis, though 3D-EVs demonstrate enhanced therapeutic potential.

**Fig. 4:**
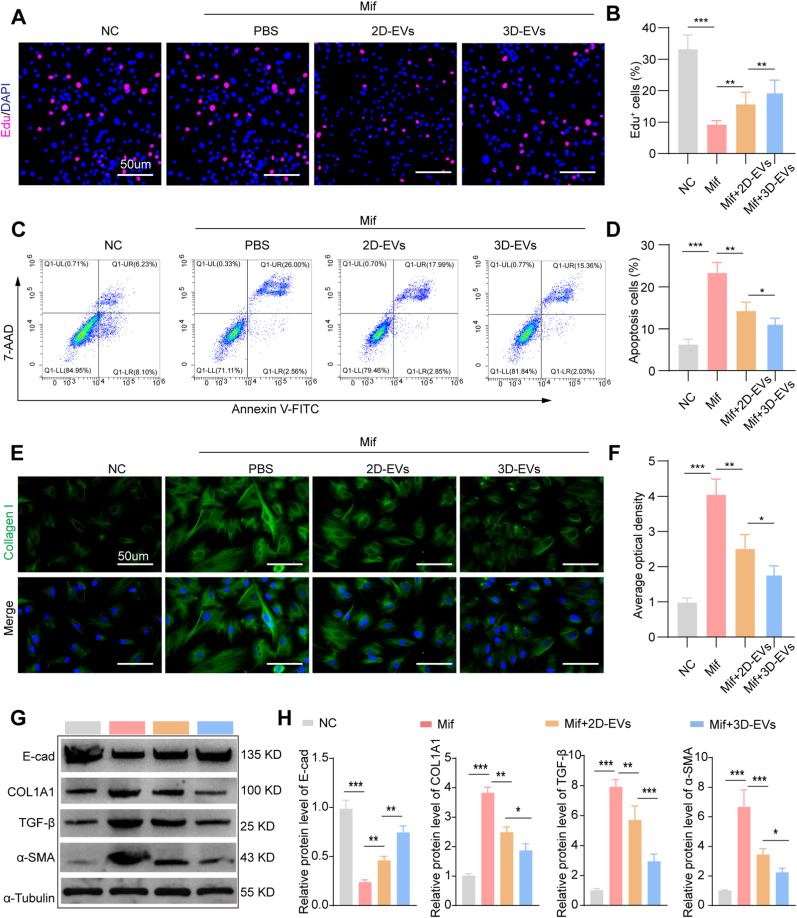
**3D-EVs protect against mifepristone-induced inhibition of hESC proliferation and alleviate fibrosis.** A, B Images of EdU assay for injury hESCs [proliferating cells (EdU^+^, red) and nuclei (DAPI, blue)] and quantitative of EdU^+^ cells, n = 5. C, D. Images of flow cytometry apoptosis analysis for injury hESCs using Annexin V-FITC/7-AAD and apoptosis cell quantification, n = 5. E, F. Images of immunofluorescence analysis of the collagen I expression of injury hESCs (green represents collagen I, and blue represents nuclei) and collagen quantification, n = 5. G, H. Western blot analysis of the protein expression levels of E-cad, COL1A1, TGF-β, and ɑ-SMA in Mif-injured hESCs with different interventions and their corresponding quantitative results, n = 3. The data represent means ± SD. E-cad: E-cadherin. (For interpretation of the references to colour in this figure legend, the reader is referred to the Web version of this article.)

The above results suggested that both 2D-EVs and 3D-EVs have excellent cell compatibility and can significantly restore the proliferation, migration, fibrosis, and apoptosis of injured hESCs. Collectively, 3D-EVs presented significant superior therapeutic potential in rescuing Mif-induced cytotoxicity.

### 3.4 Characterization of EVs@GelMA hydrogels

Given their rapid *in vivo* clearance, GelMA hydrogel was employed as a sustained-release carrier for EVs, aiming to enhance their local retention and enable effective *in situ* treatment. As illustrated in Fig. 5A and B, the preparation of EVs-loaded GelMA hydrogels involves a phase transition from liquid to solid state after being exposed to UV light irradiation for 30 s. The mechanical property test data of the GelMA hydrogel shown in Fig. S4. Fig. 5C illustrates that the GelMA solution can be readily administered through a 1 mL syringe needle, suggesting its superior plasticity and injectability. These properties enable GelMA to fill and conform to wound contours. Fourier-transform infrared spectroscopy (FTIR) analysis revealed that the chemical properties of GelMA remained unchanged after mixing with EVs, with no evidence of new chemical bond formation between the two components (Fig. 5D). To investigate EVs distribution within the hydrogel, PKH26-labeled EVs were incorporated into GelMA prior to UV-induced crosslinking. Laser confocal microscopy confirmed that EVs were uniform dispersed within the GelMA matrix (Fig. 5E and F). Given the importance of porous structure for cell adhesion, proliferation, and nutrient exchange, the microstructure of EVs-GelMA hydrogel was characterized. As shown in Fig. 5G–J, EVs incorporation did not significantly alter the hydrogel's pore architecture; GelMA and EVs@GelMA had comparable average pore sizes (100 μm) and porosity (approximately 50%).

**Fig. 5:**
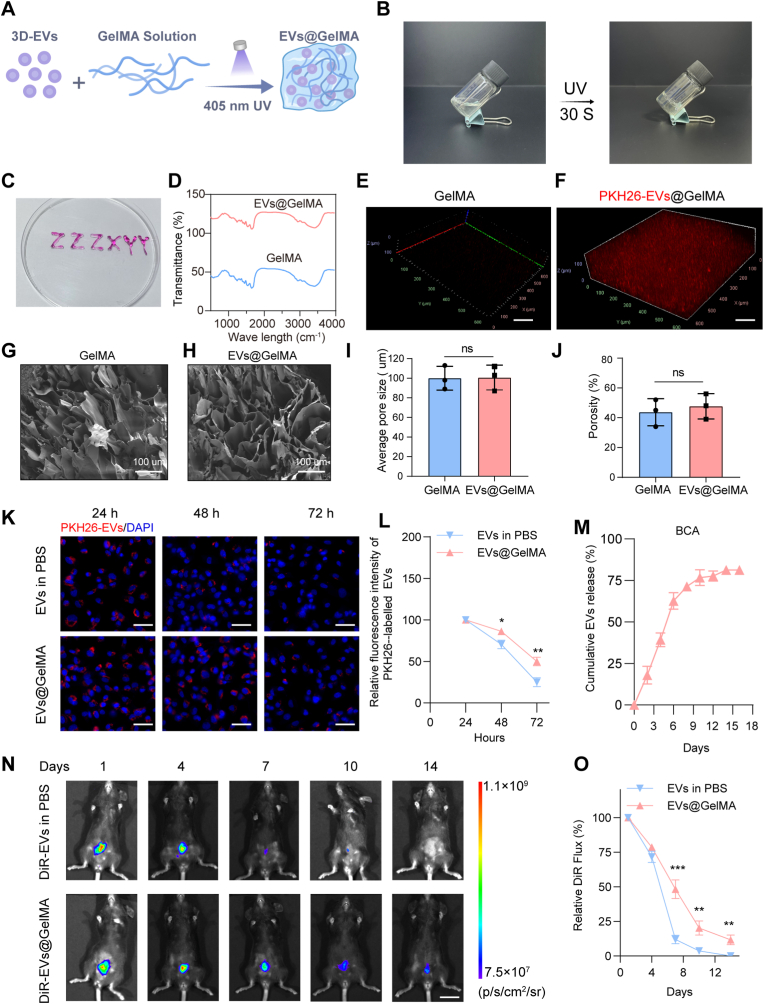
**Characterization of EVs@GelMA.** A. The process for EVs@GelMA preparation. B. Changes in the GelMA before and after UV light exposure. C. GelMA writing letters with a syringe. D. FTIR of GelMA and 3D-EVs@GelMA. E, F. Distribution of PKH26-labeled EVs in the hydrogel. G, H. TEM images for GelMA and EVs@GelMA. I, J. Pore size and porosity of GelMA and EVs@GelMA. K, L. Fluorescence image and intensity of the sustained EVs labeled with PKH26. M. BCA method assessment of EVs release kinetics of EVs@GelMA. N, O. *In vivo* images and relative DiR flux of detection of EVs retention time in the uterus. ns, not significant versus the indicated group. The data represent means ± SD, n = 3 per group, two-way repeated measures ANOVA.

Subsequently, PKH26-labeled EVs were encapsulated within GelMA hydrogel to evaluate their release kinetics. As illustrated in Fig. 5K and L, EVs were efficiently internalized by hESCs and localized in the cytoplasm after 24 h of incubation. Notably, fluorescence intensity within cells markedly declined at 48 and 72 h in both groups. However, the EVs@GelMA group demonstrated a significant higher rate of endocytosis compared to the free-EVs group at these timepoints, with the decrease in the fluorescence signal of PKH26 reduced, indicating GelMA enhanced EVs retention and sustained release. To further characterize the sustained release profile, the concentration of protein released from EVs@GelMA was quantified using BCA assay. As depicted in Fig. 5M, proteins continuously released from the hydrogel over 9 days, and by the 10th day, the cumulative release amount reached 80%; the burst release amount in the first 2 days was limited to approximately 20% of total content.

For *in vivo* evaluation, DiR-labeled EVs were incorporated into GelMA hydrogel (DiR-EVs@GelMA), and their uterine retention was assessed via fluorescence imaging. Fig. 5N demonstrates prolonged uterine retention of GelMA-encapsulated EVs, exhibiting 6.8-fold higher fluorescence intensity than free EVs in PBS at day 7 (*p* < 0.001). Quantitative analysis revealed a 10% residual EVs signal in the EVs@GelMA group at day 14, whereas free EVs showed complete clearance. These findings collectively indicate that GelMA hydrogel facilitates sustained EVs release both *in vitro* and *in vivo*, establishing its potential as an effective delivery scaffold for prolonged therapeutic intervention.

### 3.5 3D-EVs@GelMA present better therapeutic capacity in endometrial injury than 2D-EVs@GelMA *in vivo*

To further assess the therapeutic potential of 2D-EVs@GelMA and 3D-EVs@GelMA in endometrium injury, murine models of endometrial injury were established via mechanical scraping followed by ethanol perfusion. Pathological features of the endometrium were detected at different time points after injury to verify the successful construction of the mice IUA model. As shown in Fig. S5, the endometrial thickness and the number of endometrial glands were significant decreased from 7 days after injury, while the expression levels of Collagen-Ⅰ and α-SMA were significant increased from 7 days after injury. These findings confirmed that the IUA model was successfully established at 7 days after injury. Therefore, 7 days after injury, the EVs@GelMA hydrogel was injected at the lesion site and photopolymerized under 405 nm UV irradiation (designated Day 0). The experimental timeline is illustrated in Fig. 6A. Uterine morphology was evaluated 14 days post-treatment. As demonstrated in Fig. 6B, uteri in the sham-operated group maintained normal architecture and tissue elasticity. In contrast, the IUA model group exhibited severe adhesions, structural distortion, and pallor. Both 2D-EVs@GelMA and 3D-EVs@GelMA treatments significantly ameliorated these pathological changes, with 3D-EVs@GelMA demonstrating superior efficacy in restoring uterine histoarchitecture.

**Fig. 6:**
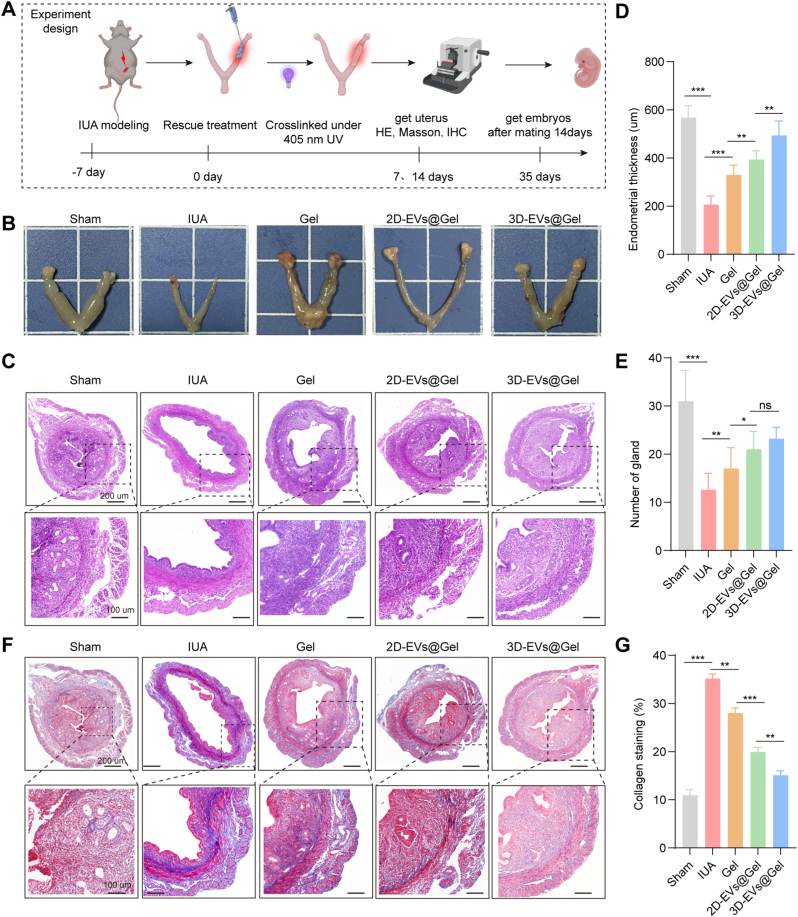
**3D-EVs@GelMA promotes endometrial repairment and alleviates collagen deposition.** A. Experimental flowchart. B. Murine uterine appearance of different groups 14 days post-treatment. C. HE staining analysis of endometria of mice in different groups 14 days after treatment. D, E. Quantitative analysis of endometrial thickness and gland. F. Masson's trichrome staining analysis of collagen deposition of the endometria of mice in different groups 14 days after treatment (blue staining). G. Quantitative analysis of collagen deposition area. ns, not significant versus the indicated group. The data represent means ± SD, n = 5 independent biological replicates. (For interpretation of the references to colour in this figure legend, the reader is referred to the Web version of this article.)

Histological analysis was performed on endometrial tissues collected 14 days post-treatment using HE staining. Representative images are presented in Fig. 6C–E. The sham group exhibited intact endometrial architecture with distinct endometrial-myometrial boundaries, characterized by columnar epithelial lining of the uterine cavity and well-developed glandular structures. These glands, predominantly round or oval in morphology, were mainly located in the endometrial stroma. In contrast, the IUA model group demonstrated complete disruption of endometrial integrity, featuring marked uterine cavity dilation, substantial glandular depletion, and significant endometrial thinning, confirming successful establishment of the IUA models. Therapeutic intervention with either 2D-EVs@GelMA or 3D-EVs@GelMA significantly improved endometrial morphology. Quantitative analysis revealed that the 3D-EVs@GelMA treatment produced superior restorative effects in increasing endometrial thickness by 2.4-fold compared to the IUA group (*p* < 0.001) and restoring glandular density to 80% of sham levels. These outcomes significantly exceeded the therapeutic efficacy of the 2D-EVs@GelMA and GelMA-only treatments, highlighting the merit of 3D-EVs in tissue regeneration.

Masson's trichrome staining (Fig. 6F and G) demonstrated extensive collagen deposition (blue staining) in IUA group endometria, confirming pathological fibrosis progression. While GelMA-only treatment showed moderate antifibrotic activity, both EVs-loaded hydrogels significantly attenuated collagen accumulation. The 3D-EVs@GelMA formulation exhibited particularly robust antifibrotic effects, reducing fibrotic collagen content by 58% versus the IUA group and nearly restoring the physiological collagen distribution pattern observed in sham specimens.

Subsequently, immunofluorescence analysis was performed to evaluate endometrial fibrosis by detecting fibrotic markers. As illustrated in Fig. 7A–D, compared with the sham group, the IUA group exhibited significantly elevated expression levels of COL1A1 (red fluorescence) and α-SMA (green fluorescence). In contrast, both EVs@GelMA treatment groups showed substantial reductions in these fibrotic markers, with the 3D-EVs@GelMA group exhibiting expression levels nearly comparable to the control group. These results indicate that 3D-EVs@GelMA possesses superior efficacy in mitigating collagen deposition and suppressing IUA-induced fibrosis in mice.

**Fig. 7:**
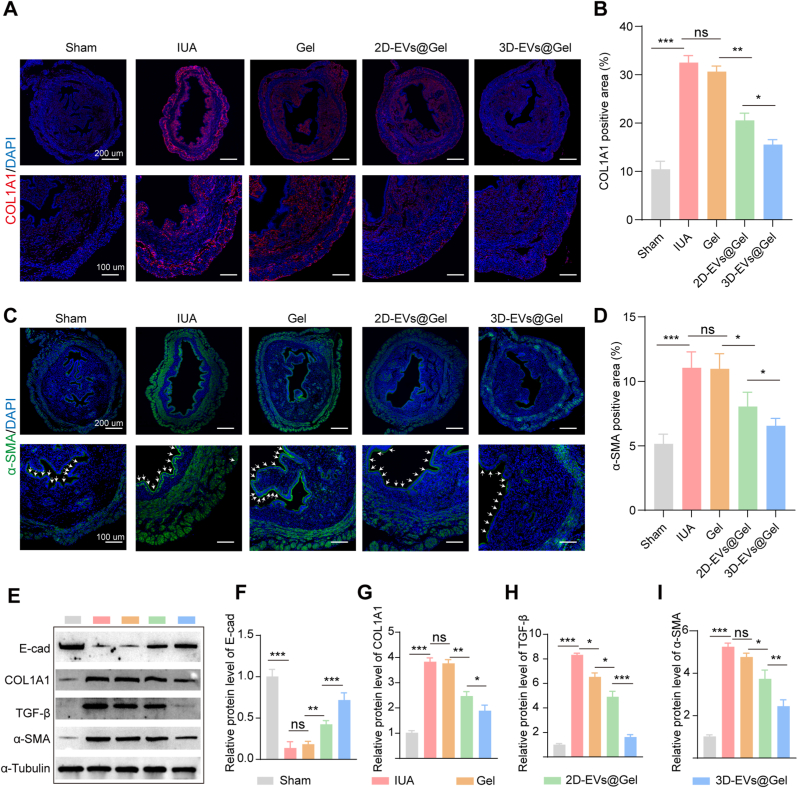
**3D-EVs@GelMA alleviated collagen deposition and inhibited fibrosis.** A, B. COL1A1 (red fluorescence) immunofluorescence results and statistical graphs. C, D. α-SMA (green fluorescence) immunofluorescence results and statistical graphs. Blue fluorescence was nuclear stained by DAPI. E, F, G, H, I. Western blot analysis of the protein expression of E-cad, COL1A1, TGF-β, and α-SMA in different treatment groups and their corresponding quantitative data. ns, not significant versus the indicated group. The data represent means ± SD, n = 5 independent biological replicates. (For interpretation of the references to colour in this figure legend, the reader is referred to the Web version of this article.)

Western blot analysis corroborated these findings at the protein level. The IUA group displayed significantly decreased E-cad expression along with markedly increased levels of COL1A1, TGF-β1, and α-SMA compared to the sham group. However, the EVs@GelMA treatment groups exhibited a significant reversal of these effects, showing increased E-cad expression and decreased levels of fibrotic markers (COL1A1, TGF-β1, and α-SMA) (Fig. 7E–I). The 3D-EVs@GelMA group demonstrated more pronounced therapeutic effects than the 2D-EVs@GelMA group, which supported its enhanced capability in attenuating collagen deposition and fibrosis progression.

### 3.6 3D-EVs@GelMA recovers fertility attenuated by endometrial injury

**Fig. 8:**
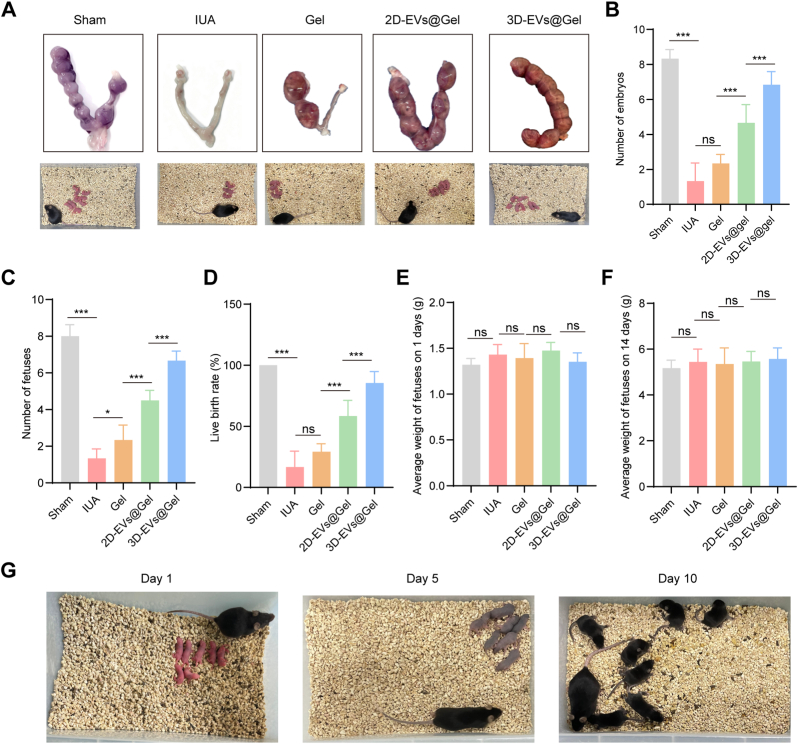
**Pregnancy results from various interventions.** A. Embryo images from the groups. B. Count of embryos in each group; n = 6. C. Count of fetuses in each group; n = 6. D. Birth rates of live offspring in various groups; n = 6. E. Average fetal weight quantified on day 1; n = 6. F. Average fetal weight quantified on day 14; n = 6. G. Images of pups in the 3D-EVs@GelMA group after 10 days of feeding. ns, not significant versus the indicated group. The data represent means ± SD.

The effects of 3D-EVs on fertility were evaluated three estrous cycles post-treatment. As illustrated in Fig. 8A and B, the IUA model group demonstrated only one embryo implantation, significantly fewer than the eight implantations observed in the sham group (*p* < 0.05). Both 2D-EVs@GelMA and 3D-EVs@GelMA treatments significantly increased implantation numbers compared to the IUA and GelMA-only groups, with the 3D-EVs@GelMA group showing the highest implantation count (*p* < 0.001 vs. all other treatment groups), indicating superior restoration of endometrial receptivity. Reproductive outcomes were further assessed through fetal parameters across treatment groups. The IUA model group exhibited significantly reduced fetal numbers and birth rates compared to sham controls. Both 2D-EVs@GelMA and 3D-EVs@GelMA treatments significantly improved these parameters, with the 3D-EVs@GelMA group demonstrating the highest fetal counts and birth rates (Fig. 8C and D). Notably, no significant differences were observed in neonatal weights at postnatal days 1 and 14 among any groups (Fig. 8E and F), suggesting that EVs@GelMA promotes fertility restoration without affecting offspring development. These findings collectively demonstrate that 3D-EVs@GelMA exhibits superior therapeutic efficacy in fertility recovery compared to 2D-EVs@GelMA, and the treatment shows promising safety profiles.

### 3.7 Transcriptome sequencing

**Fig. 9:**
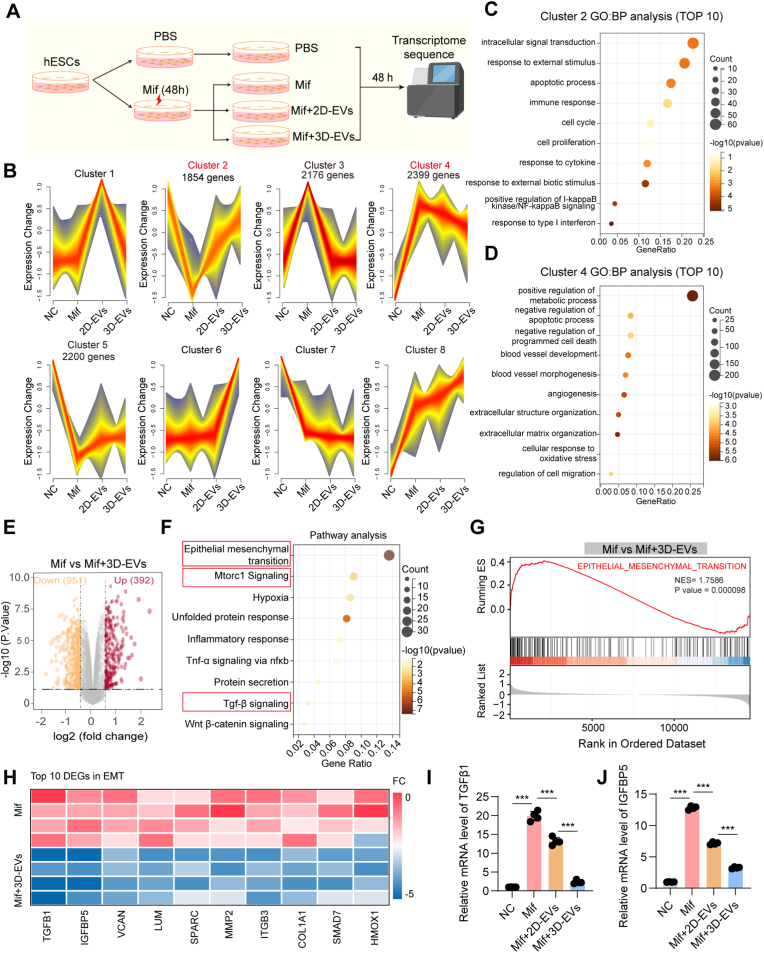
**Transcriptome sequencing results.** A. Workflow for transcriptome sequencing, n = 4. B. Gene clusters analysis using the Mfuzz clustering algorithm. C. GO-BP analysis for the 10 genes with the greatest expression differences in cluster 2. D. GO-BP analysis for the 10 genes with the greatest expression differences in cluster 4. E. Differential gene expression data between groups visualized by volcano plots. F. KEGG pathway enrichment analysis of the DEGs. G. EMT pathway GSEA. H. The top ten differentially expressed genes (DEGs) enriched in the EMT pathway are illustrated. I, J. RT-qPCR was conducted to verify the mRNA expression levels of *TGFβ1* and *IGFBP5* in hESCs with different treatments. The data represent means ± SD, n = 4 independent biological replicates.

To investigate the molecular mechanisms responsible for the enhanced therapeutic effects of 3D-EVs in endometrial injury repair, we conducted transcriptome sequencing analysis of Mif-injured hESCs following EVs treatment (Fig. 9A). Using the Mfuzz algorithm, co-expression analysis identified eight distinct temporal trajectories of mRNA expression (Fig. 9B). Genes in cluster 2 exhibited downregulation following Mif-induced injury in hESCs. Subsequently, these genes showed a gradual upregulation after treatment with 2D-EVs and 3D-EVs. Analysis of the top 10 differentially expressed genes (DEGs) using GO-BP revealed intracellular signal transduction, response to external stimuli, apoptotic process, immune response, and cell proliferation were significantly enriched (Fig. 9C). By contrast, genes in cluster 4 showed upregulation in hESCs following Mif treatment. Subsequently, these genes showed a gradual downregulation after treatment with 2D-EVs and 3D-EVs. The top 10 DEGs in cluster 4 were predominantly enriched in pathways related to: positive regulation of metabolic regulation pathways, negative regulation of apoptotic process, blood vessel development and morphogenesis, angiogenesis and regulation of cell migration (Fig. 9D). These temporally correlated expression dynamics of the two gene clusters with EVs intervention may represent critical molecular signatures contributing to the superior therapeutic efficacy of 3D-EVs compared to 2D-EVs.

Volcano plot analysis of DEGs between the Mif-only group and Mif-3D-EVs-treated group revealed significant transcriptional changes, with 392 genes upregulated and 951 genes downregulated following 3D-EVs treatment (Fig. 9E). Subsequent KEGG pathway enrichment analysis of these DEGs demonstrated significant enrichment in multiple pathways, including epithelial-mesenchymal transition (EMT), mTORC1 signaling, inflammatory responses, protein secretion, and TGF-β signaling (Fig. 9F). In particular, EMT-related pathways suggested the potential involvement in 3D-EVs-mediated repair mechanisms. Based on these findings, we further conducted a targeted gene set enrichment analysis (GSEA) focusing on the EMT signaling pathway. The results revealed a statistically significant downregulation of EMT-related genes in the 3D-EVs-treated group, with a normalized enrichment score of 1.7586 and a *p*-value of 0.000098 (Fig. 9G). The top 10 DEGs enriched in the EMT pathway are presented in Fig. 9H, with distinct expression patterns observed between the Mif and Mif-3D-EVs groups. To verify the transcriptome sequencing data, RT-qPCR was performed on two of the most significantly regulated genes: *TGFβ1* and *IGFBP5*. The mRNA expression levels of these two genes were significantly downregulated in the Mif-3D-EVs group compared with the Mif group, which was consistent with the RNA-sequencing results (Fig. 9I and J). These findings validate the reliability of the transcriptomic data and provide robust evidence supporting that 3D-EVs negatively regulate EMT processes during the repair of injured hESCs.

### 3.8 3D-EVs May deliver BECN1 to inhibit fibrosis and accelerate endometrial repair

**Fig. 10:**
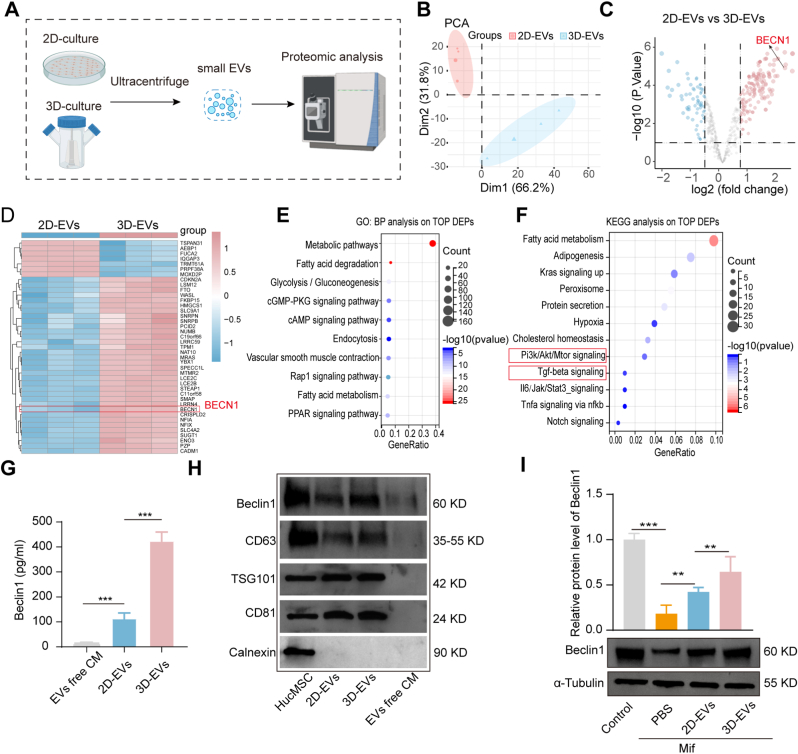
**Proteome analysis for the mechanism by which 3D-EVs have superior treatment potential.** A. Experimental design,n = 3. B. PCA illustrated the protein expression patterns of 3D-EVs and 2D-EVs. C. Volcano plot analysis of the differential expression proteins of 3D-EVs and 2D-EVs. D. Heatmap of the top 40 differentially abundant proteins in 3D-EVs vs. 2D-EVs. E. GO-BP analysis for the 40 genes with the greatest expression differences. F. KEGG analysis for the 40 genes with the greatest expression differences. G. ELISA analysis of the expression levels of BECN1 in 2D-EVs and 3D-EVs. H. Western blot analysis the levels of proteins BECN1, CD63, TSG101, and CD81 in 2D-EVs and 3D-EVs. I. Western blot analysis and quantitative analysis of the levels of protein BECN1 in Mif-injured hESCs with NC, 2D/3D EVs treatments. The data represent means ± SD, n = 3 independent biological replicates.

To clarify the mechanism by which 3D-EVs exert better therapeutic advantages, we employed LC-MS/MS proteomic analysis of 2D-EVs and 3D-EVs (Fig. 10A). PCA revealed distinct clustering of 2D-EVs and 3D-EVs, indicating fundamental proteomic differences (Fig. 10B). Volcano plot analysis identified 127 significantly dysregulated proteins (|log_2_FC| ≥ 1, *p* < 0.05) (Fig. 10C), among which the autophagy-related protein BECN1 was upregulated in the 3D-EVs group. A heatmap displaying the top 40 differentially expressed proteins (DEPs) in 3D-EVs compared with 2D-EVs further verified the upregulation of BECN1 (Fig. 10D). The top 40 DEPs were then selected for GO-BP and KEGG analysis. We found that the DEPs are associated with metabolic, cGMP-PKG signaling, CAMP signaling pathway and fatty acid metabolism, adipogenesis, adipogenesis, hypoxia, PI3k/Akt, and TGF-β signaling (Fig. 10E and F). These analyses may represent distinct cellular functions unique to 3D-EVs versus 2D-EVs.

As BECN1 reduces fibrosis and promotes cell regeneration in various diseases, we further validated the expression of BECN1 in 3D-EVs. ELISA and Western blot results demonstrated that the expression level of BECN1 in 3D-EVs was three-fold higher than in 2D-EVs, and both were significantly higher than in EVs-free cell supernatant (*p* < 0.001) (Fig. 10G and H), which was consistent with the volcano plot and heatmap analysis. Furthermore, Western blot analysis was conducted to investigate the effects of 2D-EVs and 3D-EVs on BECN1 expression in Mif-injured hESCs. Results indicated that 3D-EVs treatment induced a more marked upregulation of BECN1 in hESCs relative to 2D-EVs (Fig. 10I), implying that BECN1 may contribute substantially to the enhanced therapeutic efficacy of 3D-EVs.

### 3.9 Inhibition of BECN1 partly abolished the therapeutic effects of 3D-sEVs in the reversal of IUA fibrosis

To clarify whether the superior therapeutic efficacy of 3D-EVs in treating IUA is attributed to the delivery of BECN1, we knocked down BECN1 expression in HucMSCs and thereby obtained 3D-EVs with reduced BECN1 concentration (3D-EVs^sh-Beclin1^). Fig. S7A illustrates the systematic experimental strategy adopted to explore the role of BECN1 in HucMSCs and their secreted EVs. Fig. S7B shows the typical spindle-shaped morphology of HucMSCs, while the fluorescent micrograph (Fig. S7C) demonstrates efficient green fluorescent protein (GFP) expression, which confirms the successful transduction of lentivirus into HucMSCs. Subsequently, the efficiency of BECN1 knockdown in HucMSCs was verified via RT-qPCR and Western blot analysis. As presented in Figs. S6 and S7D, the results reveal a significant decrease in BECN1 protein levels in HucMSCs treated with sh-Beclin1-3 compared to those in the shNC control group, confirming the successful knockdown of BECN1. Fig. S7E–F show that no significant morphological differences were observed between EVs derived from sh-NC (3D-EVs^sh-NC^) and 3D-EVs^sh-Beclin1^, and their size distribution and concentration were also comparable. These findings suggest that BECN1 knockdown does not affect the basic structural characteristics of EVs. Fig. S7G indicates that both 3D-EVs^sh-NC^ and 3D-EVs^sh-Beclin1^ express the EV-specific markers CD81, CD63, and TSG101, while being negative for Calnexin. The expression of these specific markers validates the identity and purity of the isolated particles as EVs. Finally, Fig. S7H displays the ELISA results comparing BECN1 levels in HucMSCs and their secreted EVs under sh-NC and sh-BECN1 conditions. A significant reduction in BECN1 levels was detected in both HucMSCs^sh−Beclin1^ cells and their secreted 3D-EVs^sh-Beclin1^ compared to the control group. This observation highlights the effective knockdown of BECN1 and its corresponding reflection in the secreted EVs, implying that BECN1 is actively encapsulated into EVs and that its levels can be regulated through cellular knockdown strategies.

**Fig. 11:**
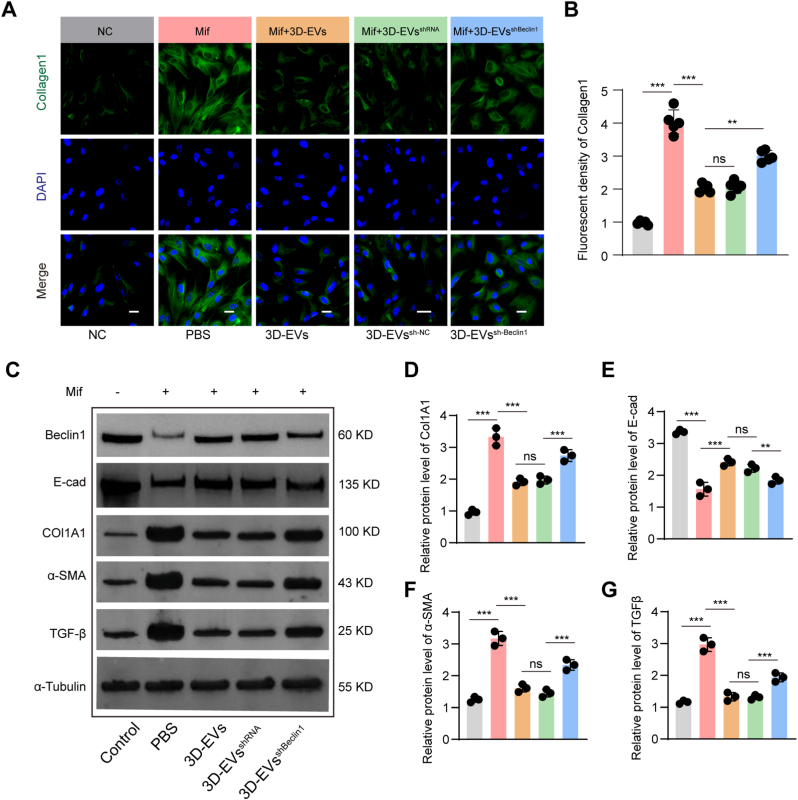
**Inhibition of BECN1 partly rescue the ameliorative effects of 3D-EVs on fibrosis in Mif-injured hESCs.** (A-B) Immunofluorescence analysis of Collagen1 (A: representative images; B: quantitative analysis), n = 5. (C) Western blot analysis the expression levels of fibrosis-related proteins (E-cad, Collagen 1, α-SMA, and TGF-β) and BECN1 in hESCs following distinct treatments. (D-G) Quantitative analysis of the Western blot results shown in (C), n = 3. ns, not significant versus the indicated group. Data are presented as means ± SD.

Subsequently, immunofluorescence staining and Western blot analysis were employed to investigate the association between BECN1 and the enhanced fibrosis-reducing capacity of 3D-EVs. Fig. 11A and B presents the immunofluorescence staining results assessing Collagen1 expression in injured hESCs after suppressing BECN1 in 3D-EVs. Compared with the Mif group, the 3D-EVs-treated group exhibited a significant increase in Collagen1 expression. However, when BECN1 in 3D-EVs was knocked down (3D-EVs^sh-Beclin1^), the stimulatory effect on Collagen1 expression was significantly weakened. Western blot analysis results showed that the protein levels of α-SMA, TGF-β, and Collagen-1 in the 3D-EVs group were significantly lower than those in the Mif group, but this difference was significantly attenuated after BECN1 knockdown. Similarly, the relative protein levels of E-cad exhibited a similar trend: 3D-EVs promoted E-cad expression, while BECN1 knockdown alleviated this promotional effect (Fig. 11C–G). These findings underscore the pivotal role of BECN1 within 3D-EVs in modulating the anti-fibrotic effects in injured hESCs.

## 4 Discussion

IUA is the second-leading cause of female infertility and pose significant threats to the physical and psychological well-being in women of reproductive age [55]. Endometrial fibrosis is hallmark pathological characteristic of IUA, which remains refractory to conventional therapeutic approaches that fail to reverse fibrotic progression, consequently leading to suboptimal endometrial receptivity and poor pregnancy outcomes [56] and prompting the development of innovative therapies. In this study, we introduced GMs to 3D culture of MSCs, which enabled high-yield production of superior-quality extracellular vesicles (3D-EVs). Subsequent evaluation demonstrated that 3D-EVs have significantly enhanced therapeutic potential for endometrial repair compared to conventional 2D-culture-derived small EVs (2D-EVs) in both *in vitro* and *in vivo* models. Mechanistic studies revealed that 3D-EVs markedly upregulated the expression of the autophagy-related protein BECN1 (Beclin-1), which potentially accounts for their improved therapeutic efficacy in endometrial regeneration.

As a promising cell-free therapy approach, EVs have garnered increasing attention in regenerative and immunomodulatory medicine. Accumulating evidence has demonstrated the therapeutic potential of MSCs-EVs for endometrial injury repair [57]. Given that the biological content and functional properties of EVs are dynamically influenced by the physiological status and culture microenvironment of parental MSCs [58], this study aimed to develop an optimized cell culture strategy to enhance both EVs production and their therapeutic efficacy against IUA. Furthermore, we investigated the underlying mechanisms to provide insights for standardizing cell culture protocols.

3D culture systems offer distinct advantages over conventional 2D cultures by better recapitulating the native tissue architecture, including cell-cell and cell-matrix interactions, while avoiding microenvironmental distortions caused by the lack of spatial organization in monolayer cultures [59]. Emerging evidence suggests that 3D MSC culture technology holds great promise for various applications, including inflammatory disease treatment, tissue regeneration, and targeted therapy while significantly improving both the yield and therapeutic potential of derived EVs [60]. Various 3D cell culture methods for producing EVs have been reported. EVs derived from hMSCs cultured using microbead-based 3D systems exhibited enhanced angiogenic and proliferative capabilities compared to their 2D counterparts, along with enhanced viability and tube-forming ability in human umbilical vein endothelial cells [16]. Similarly, MSCs-derived EVs produced by a hollow fiber 3D culture system showed both higher yield and more pronounced cardioprotective effects in acute myocardial infarction rat models when compared to 2D-cultured EVs [61]. In general, 3D cultivation technologies can be classified into two categories: scaffold-based culture systems and scaffold-free culture systems. The former offers precise control over microenvironmental parameters including stiffness and porosity, while the latter more closely mimic natural tissue development processes [62,63]. Here, we employed GMs as a scaffold-free 3D culture platform for EV production, as it has been shown to enhance cell viability, maintain pluripotency, and augment therapeutic properties including angiogenic potential, antifibrotic activity, and immunomodulatory effects [64,65]. HucMSCs produce the highest exosome yield among the common MSCs from different sources, and because they are abundant in supply and easy to obtain and have low immunogenicity and a strong proliferation ability [66,67], we selected them for EVs production. According to our results, 3D-EVs yielded significantly more than 2D-EVs. *In vitro* functional assays revealed that both 2D-EVs and 3D-EVs significantly restored proliferation, migration, viability, and collagen deposition in Mif-injured hESCs. However, the rapid clearance property of EVs *in vivo* significantly affects their retention time in the target tissue and their therapeutic effect [68], while repeated local injections increase the pain. Therefore, we used GelMA hydrogel as the EVs carrier for local treatment with excellent delivery efficiency and therapeutic durability. Consistent with previous results [69], our study confirmed that GelMA effectively extended EVs release kinetics both *in vitro* and *in vivo*. Further, we evaluated the effects of 3D-EVs@GelMA on endometrium injury repair in an IUA mouse model. We found that 2D-EVs@GelMA and 3D-EVs@GelMA significantly improved endometrial parameters, including fibrosis reduction, restoration of endometrial thickness, and increased glandular number. Notably, 3D-EVs consistently outperformed 2D-EVs in therapeutic efficacy in both *in vitro* and *in vivo* experiments, which suggests their superior potential for clinical endometrial repair applications.

It is worth mentioning that GelMA alone exhibited significant therapeutic effects on IUA, which is consistent with the findings reported in previous studies [[70], [71], [72]]. The observed therapeutic benefits may be attributed to several distinctive structural and functional properties of this hydrogel system. ⅰ) The inherent plasticity and injectability of GelMA allow it to conform optimally to the injured uterine cavity. Its 3D network structure and RGD motifs provide structural support and cell adhesion sites, which are essential for endometrial regeneration [73]. ⅱ) Excessive expression of inflammatory chemokines can lead to persistent inflammation and significantly impair wound healing [74]. GelMA can serve as a barrier to isolate danger signals, reduce related stimulation, and prevent tissues from suffering secondary injury [75]. In addition, this hydrogel may also capture inflammatory cytokines through electrostatic interactions with these molecules [76]. ⅲ) As a semi-natural hydrogel, GelMA contains components similar to the *in vivo* ECM, thereby retaining endogenous factors (e.g., growth factors and matrix metalloproteinases) that are beneficial to wound healing, morphogenesis, and tissue repair [38,77]. The sustained-release capability of GelMA enables the prolonged release of these factors to promote endometrial repair. However, the precise molecular mechanisms underlying the therapeutic potential of GelMA for IUA remain to be further systematically investigated, and its single-agent therapeutic effect is not yet fully satisfactory. To enhance the therapeutic efficacy, GelMA hydrogel is often used as a 3D delivery platform for cells, cytokines, chemicals, drugs, nanoparticles, and other therapeutic agents, enabling continuous treatment at the lesion site [[77], [78], [79], [80]]. In the present study, the therapeutic effect was significantly enhanced when GelMA was combined with EVs, particularly when used in conjunction with 3D-EVs.

Although accumulating evidence has demonstrated the superior therapeutic potential of 3D-EVs, the molecular mechanisms underlying their enhanced efficacy remain poorly understood. Our proteomic profiling revealed that culture dimensionality is a crucial determinant of EVs functional characteristics. Among the top 40 DEPs, BECN1, which was reported to have antifibrotic properties, was expressed significantly higher in 3D-EVs than in 2D-EVs, which attracted our attention. BECN1, the first identified mammalian autophagy-related protein, functions as a molecular scaffold for the class III phosphatidylinositol 3-kinase (PI3KC3) complex and plays pivotal roles in autophagy initiation and various cellular trafficking processes [81]. Emerging evidence has demonstrated that BECN1 exerts regulatory functions in fibrosis across multiple organ systems. In renal fibrosis models, BECN1 can protect against acute kidney injury, preserve cellular proliferation capacity, and inhibit fibrotic progression in kidney tubules [82]. In pulmonary fibrosis, TGF-β1 inhibits BECN1 expression by upregulating UHRF1, consequently promoting fibroblast activation and fibrotic development [83]. Similarly, in cardiac fibrosis, BECN1 modulates the fibrotic process via regulation of endothelial-to-mesenchymal transition (EndMT) [84]. Yan et al. determined that MSC-EVs may promote hepatic stellate cell ferroptosis through BECN1 delivery, identifying BECN1 as a potential therapeutic target for liver fibrosis [85]. Therefore, we suspect that BECN1 is related to the superior treatment effect of 3D-EVs on endometrium injury. To further explore the relationship between enhanced BECN1 expression and the improved therapeutic efficacy of 3D-EVs, we obtained 3D-EVs with a lower BECN1 concentration from HucMSCs with BECN1 knocked down, and then investigated their reparative effect on fibrosis in injured hESCs. Results showed that the BECNI knockdown lead to the downregulation of E-cad, as well as the upregulation of COL1A1, TGF-β1, and ɑ-SMA. E-cad, an epithelial cell marker, is downregulated during the process of fibrosis [86]. As a master regulator of fibrosis, TGF-β1 can induce the expression of fibrotic markers including COL1A1 and α-SMA [87]. These results demonstrated that BECN1 knockdown *in vitro* attenuated the antifibrotic effects of 3D-EVs, suggesting that the superior therapeutic efficacy of 3D-EVs is partially mediated via enhanced delivery of BECN1. Given that extracellular vesicles act as natural delivery vehicles that co-transport diverse bioactive components-including proteins, lipids, mRNAs, and microRNAs-to exert their biological functions, we emphasize that the therapeutic benefits observed in this study most likely arise from the combined effects of multiple functional cargoes encapsulated within EVs, rather than from a single protein or biomolecule alone.

Our RNA-sequencing analysis further identified two distinct gene clusters most related to the repair process in Mif-injured hESC. They mainly enriched biological processes such as immune response, cell proliferation, negative regulation of apoptotic process, blood vessel development and morphogenesis, angiogenesis, and regulation of cell migration; all are important for endometrial repair and regeneration [74]. The results indicate 3D-EVs achieve better repair effects possibly by regulating the expression of these two cluster genes in injured hESCs. DEGs between the 3D-EVs treatment group and Mif-injured group involves many signaling pathways related to endometrial damage and fibrosis, sucn as EMT, mTORC1 signaling, inflammatory responses, and TGF-β signaling [88,89]. Our GSEA and RT-qPCR results further illustrated EMT was significantly downexpressed in the 3D-EVs treatment group. EMT process contributes significantly to the development of fibrosis in various tissues, targeting EMT inhibition represents a promising therapeutic strategy for mitigating tissue and organ fibrosis [90,91]. Accumulating evidence indicates that impaired autophagy exacerbates EMT-driven fibrotic processes in the lungs, liver, and kidneys [43,92]. Upon injury stimulation, endometrial epithelial cells undergo partial EMT, lose their epithelial characteristics, and acquire a mesenchymal phenotype, thereby promoting the fibrotic process [47]. Previous studies showed in endometrial epithelial cells, pharmacological inhibition of autophagy promotes EMT and activates pro-fibrotic signaling pathways; conversely, enhancing autophagy attenuates both EMT and fibrosis, suggesting that autophagy modulates endometrial fibrogenesis by regulating EMT [93]. Mechanistically, studies have demonstrated that autophagy deficiency stabilizes Snail and Twist-two master transcriptional regulators of EMT-by impairing their degradation [94]. As a key regulatory factor in the autophagy mechanism, the core function of BECN1 is to initiate and regulate the formation of autophagosomes. Therefore, we propose that BECN1 exerts an inhibitory effect on EMT via the activation of autophagy, thereby ameliorating endometrial fibrosis. Nevertheless, this hypothesis requires further experimental validation. Notably, TGF-β signaling is concurrently enriched among the DEGs from the transcriptome and the DEPs from the proteome. Aberrant activation of TGF-β signaling promotes EMT, thereby driving the progression of fibrosis [3]. These findings suggest that TGF-β may participate in the beneficial effects of 3D-EVs during endometrial injury repair.

This study has certain limitations that should be acknowledged. First, although we have demonstrated the proof-of-concept efficacy of 3D-EVs, the underlying regulatory mechanisms by which 3D culture enhances EV functionality-specifically, its role in upregulating BECN1 expression in hucMSCs and subsequent loading into EVs-remain elusive. Elucidation of these mechanisms is a critical step to advance the technology of 3D cell culture and will be the focus of our future investigations. Second, beyond fibrosis, IUA are pathologically complex, involving angiogenesis and immune cell regulation. Our current study did not cover these aspects, which we plan to address in future research work. Additionally, we did not evaluate long-term safety or perform a systematic dose-response analysis for 3D-EVs@GelMA. Prior to clinical translation, comprehensive safety assessments (including potential immune reactions and tumorigenic risks) and optimization of the dose-response relationship are indeed warranted. Finally, all experiments in this study were conducted in a mouse model. Given the inherent pathological differences between rodents and humans, the potential challenges regarding clinical translation of our findings require further validation in more clinically relevant models.

## 5 Conclusion

Our study demonstrates that compared to conventional 2D-cultured EVs, EVs derived from HucMSCs cultured in GMs exhibit superior therapeutic efficacy in ameliorating Mif-induced hESC injury and mechanical injury-induced IUA in mice. This therapeutic effect is partially mediated by delivering increased levels of BECN1 to injured cells. Collectively, these findings establish a new avenue for nonhormonal endometrial repair strategies targeting IUA, offering significant clinical translational potential.

## CRediT authorship contribution statement

**Juan Peng:** Conceptualization, Data curation, Funding acquisition, Methodology, Writing – original draft. **Juan Zhang:** Formal analysis, Investigation, Methodology, Resources. **Qingzhao Li:** Funding acquisition, Investigation, Methodology, Software. **Weijun Liu:** Methodology, Resources, Software. **Wenda Zou:** Data curation, Formal analysis, Investigation, Methodology. **Liyu Zhu:** Investigation, Methodology, Software. **Qianyin Zhou:** Data curation, Investigation, Methodology. **Dan Liu:** Data curation, Methodology, Software. **Man Jia:** Data curation, Formal analysis, Methodology. **Hui Li:** Conceptualization, Project administration, Supervision, Writing – review & editing.

## Declaration of competing interest

The authors declare the following financial interests/personal relationships which may be considered as potential competing interests: Juan Peng reports financial support was provided by Hunan Provincial Natural Science Foundation of China. Qingzhao Li reports financial support was provided by Hunan Provincial Natural Science Foundation of China. Juan Peng reports financial support was provided by Health Research Project of Hunan Provincial Health. If there are other authors, they declare that they have no known competing financial interests or personal relationships that could have appeared to influence the work reported in this paper.

## Figures and Tables

**figs1:**
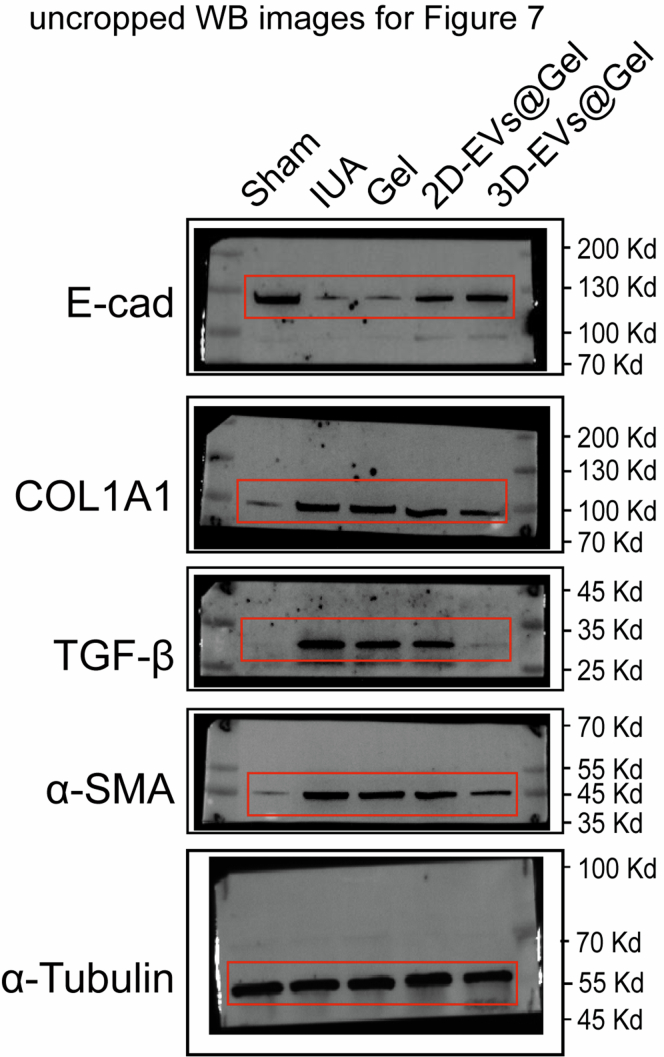


**figs2:**
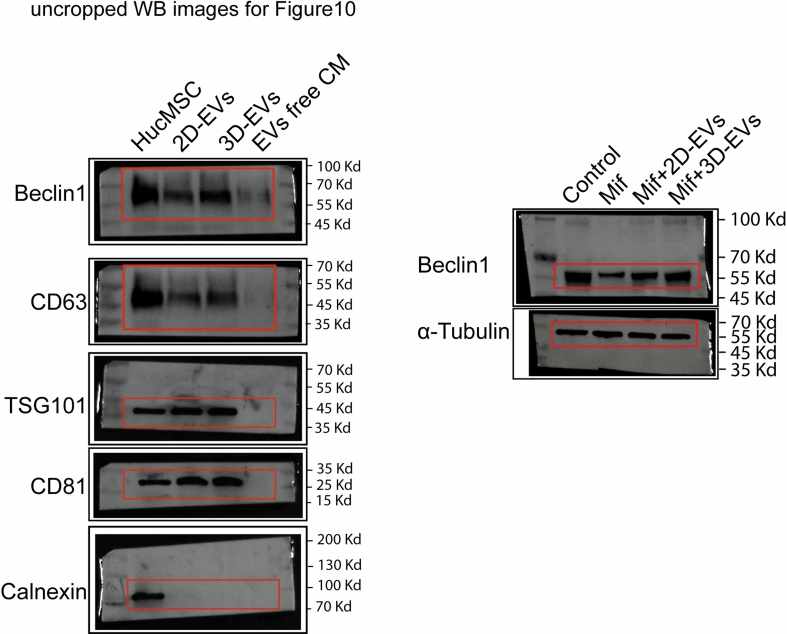


**figs3:**
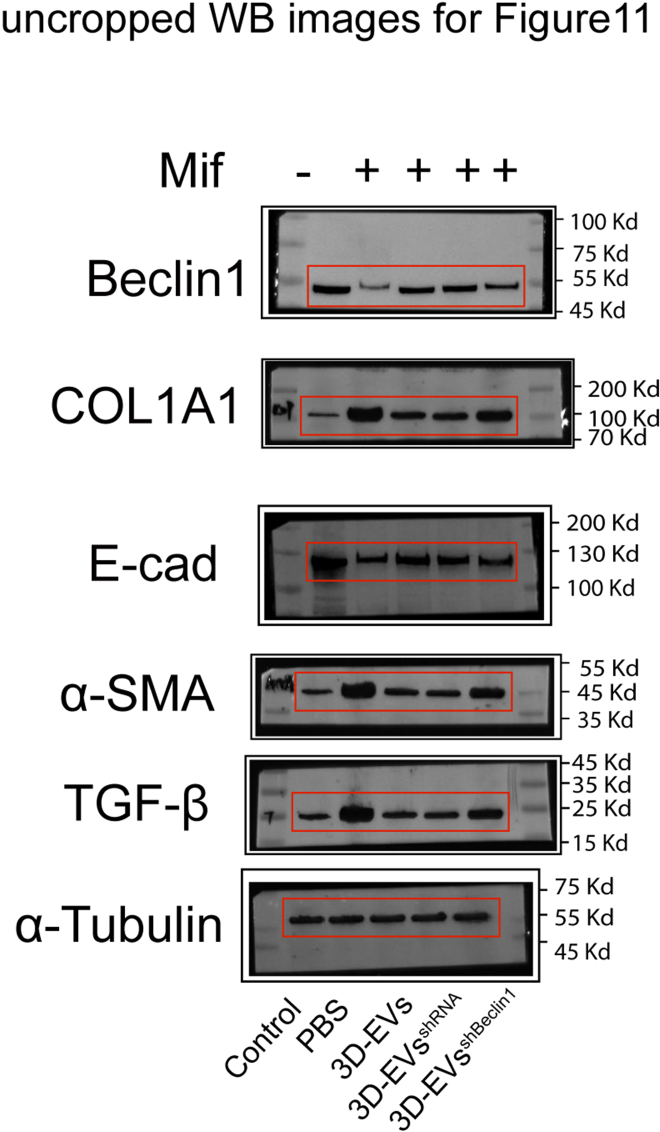


**figs4:**
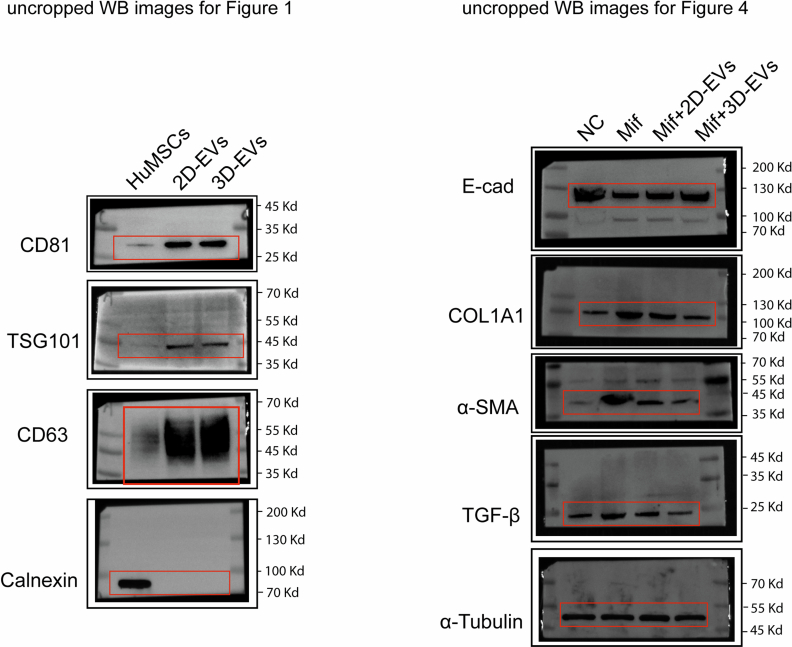


**figs5:**
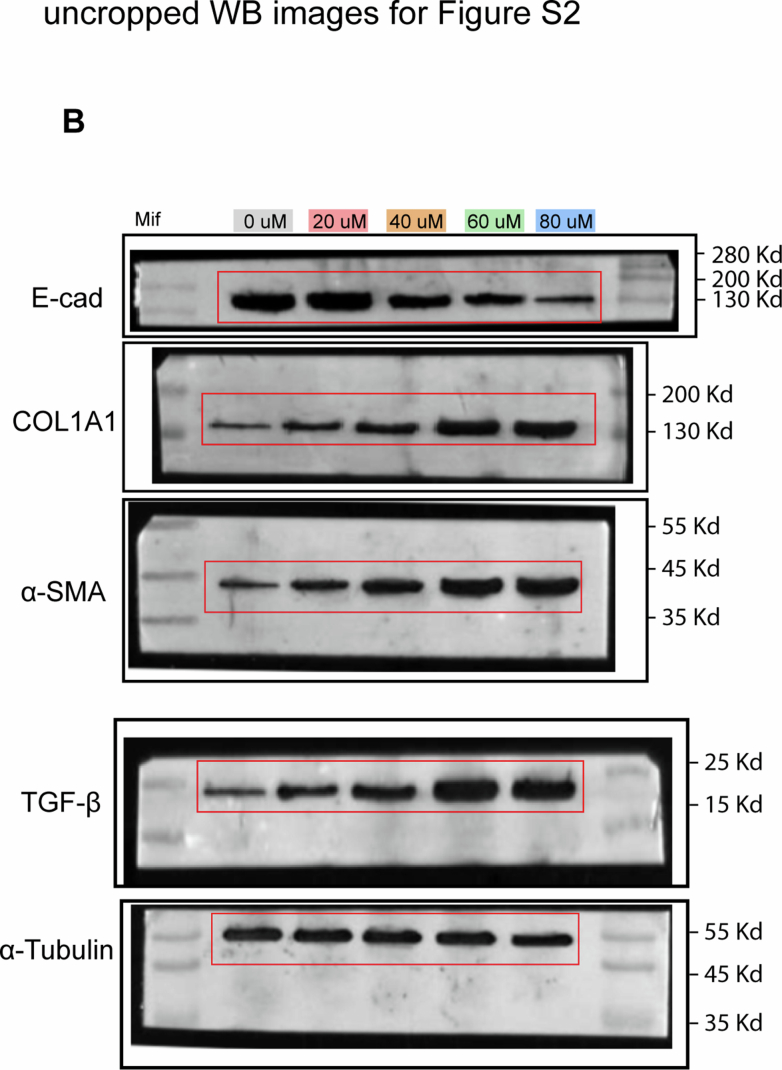


**figs6:**
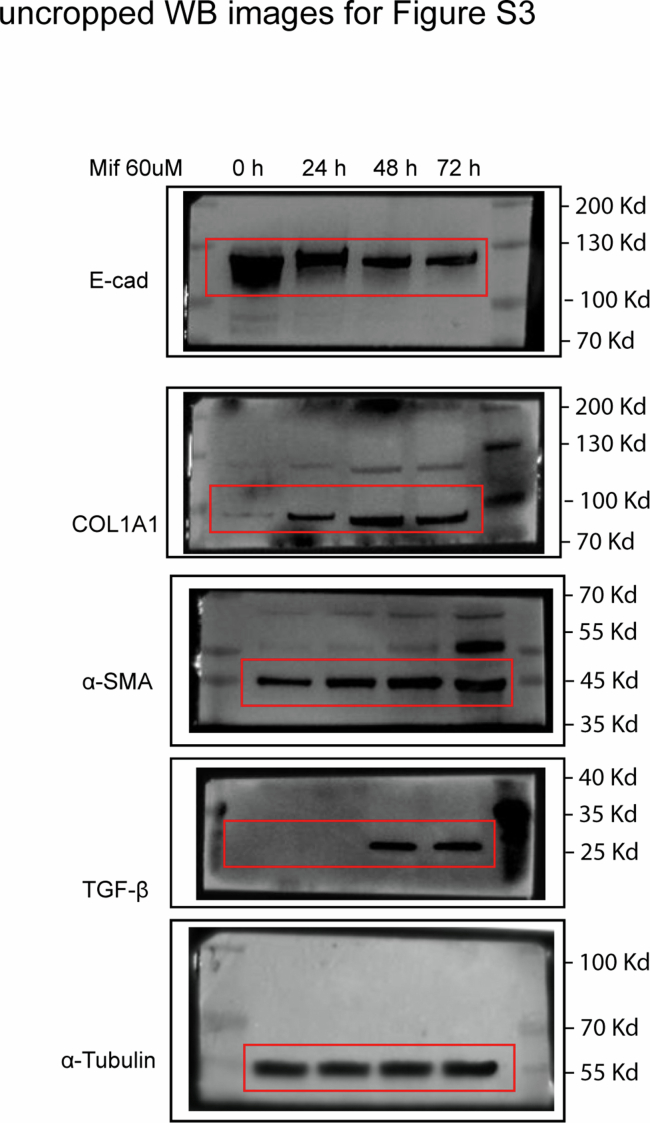


**figs7:**
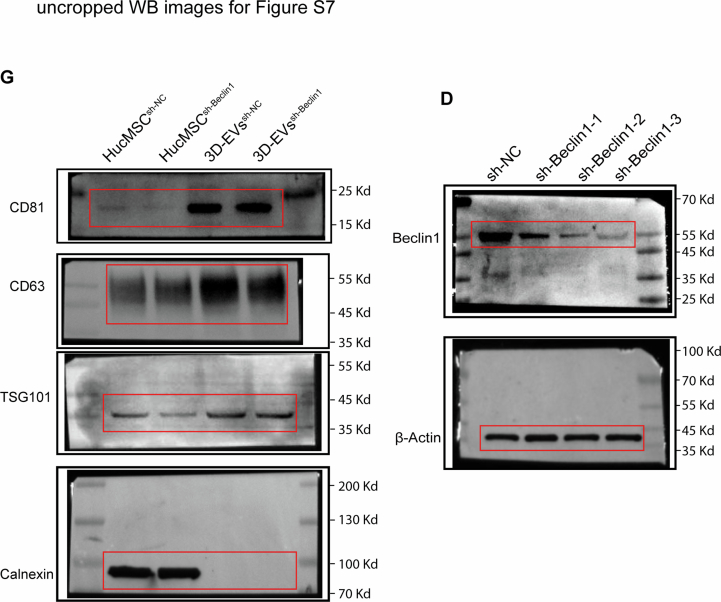


## Data Availability

Data will be made available on request.
