# Supplementary material for: 3D HucMSCs derived extracellular vesicles enhanced therapeutic efficacy in treating intrauterine adhesions via BECN1 delivery
Source: Mater Today Bio. 2026 May 29;38:103276. doi: 10.1016/j.mtbio.2026.103276 (PMC13241656; doi:10.1016/j.mtbio.2026.103276)
Supplement: Multimedia component 1 [file mmc1.doc]

# Title: 3D HucMSCs Derived Extracellular Vesicles Enhanced Therapeutic Efficacy in Treating Intrauterine Adhesions via BECN1 Delivery

# Juan Peng^1^, Juan Zhang^1^, Qingzhao Li^3^, Weijun Liu^1^, Wenda Zou^1^, Liyu Zhu^1^, Qianyin Zhou^1^, Dan Liu^1^, Man Jia^1^, Hui Li^2^*

1. Reproductive and Genetic Medicine Department, Zhuzhou Hospital Affiliated to Xiangya School of Medicine, Central South University. Zhuzhou，Hunan, China.

2. Translational Medicine Center, Zhuzhou Hospital Affiliated to Xiangya School of Medicine, Central South University. Zhuzhou，Hunan, China.

3. Hematology department, Zhuzhou Hospital Affiliated to Xiangya School of Medicine, Central South University. Zhuzhou, Hunan, China.

*Corresponding author: Hui Li. Email: lihui_1973@126.com.

**1. Supplementary Methods**

- 1. **Fluorescence labeling of EVs**

To monitor the internalization of EVs, isolated EVs (1×10^10^ particles) were labeled with PKH26 (Sigma, USA) through incubation at 37°C for 30 minutes. PKH26-labeled EVs were subsequently purified via ultracentrifugation (100,000 × g, 120 min). hESCs were seeded in confocal dishes and incubated with labeled EVs for 24 h, washed three times with PBS and fixed with 4% paraformaldehyde. Then, the cells were stained with FITC-phalloidin (Solarbio, China) and DAPI (Thermo Fisher, USA). The uptake of EVs was observed via confocal laser scanning microscopy (Carl Zeiss, Germany).

- 1. **Wound healing and Cell migration assays**

For the wound healing assay, injured hESCs were seeded into a 6-well plate. A 200 µl pipette was was employed to create a scratch of uniform width in each well, which was then marked and photographed using an inverted microscope. PBS was added to gently rinse the wells 2-3 times. Subsequently, 2D-EVs and 3D-EVs were introduced into the culture flask. The flask was then incubated at 37°C. After 48 hours of incubation, changes in the scratch were observed under an inverted microscope, and images were captured at the designated control marking points.

In the transwell assay, following the intervention with EVs, hESCs from each experimental group were introduced into the upper chamber containing serum-free DMEM. The lower chamber was supplemented with DMEM containing 10% FBS. After an 8-hour incubation, the migrating cells were fixed and stained with crystal violet. Photographs were taken and the cells were counted.

- 1. **Cell viability and live/dead assays**

For the EdU assay, cells from each group were seeded in 24-well plates. Once the cells reached 70-80% confluence, EdU (Thermo Fisher, USA) was added and incubated at 37°C for 2 hours. Subsequently, the cells were fixed and stained with the Click reaction mixture, and the nuclei were counterstained.

The CCK8 assay was used to assess the proliferation of hESCs. Injured hESCs, with/without EVs intervention, were seeded into 96-well plates, and the CCK-8 reagent was added following cell adhesion. After incubation at 37℃ for 1 h, absorbance (450 nm) was measured.

The survival and growth of the injured hESCs in each group was measured using a Live/Dead cell staining kit. Briefly, cells was rinsed with PBS. followed by the addition of 8 µM propidium iodide and 2 µM calcein-AM, and incubated at 37℃ in the dark for 30 min. After two PBS washes, the cells were observed under a fluorescence microscope to distinguish live (green) and dead (red) cells.

For the EdU assay, cells from each group were seeded in 24-well plates. Once the cells reached 70-80% confluence, EdU (Thermo Fisher, USA) was added and incubated at 37℃ for 2 h. Subsequently, the cells were fixed and stained with the Click reaction mixture, nuclei were counter stained with DAPI.

Apoptosis was assessed using the Annexin V-FITC/7-AAD apoptosis detection kit (MLBio, Shanghai, China) following the manufacturer's protocol. In brief, 5×10^5^ cells were collected and washed twice with pre-cooled PBS. Cells were resuspended by 100 μl 1× binding bufer and incubated with 5 μl of Annexin V-FITC and 10 μl of 7-AAD staining solution for 30 min in the dark at 4℃. Subsequently, 400 μl of 1× binding buffer was added, and the samples were analyzed via flow cytometry..

**1.4 Cell fibrosis assay**

Cell fibrosis was evaluated through by immunofluorescence staining. Injured hESCs, with/without EVs treatment, were fixed with 4 % paraformaldehyde for 10 min and blocked with normal goat serum at room temperature for 1 hour. The hESCs were then incubated with an anti-Collagen I antibody at 4°C overnight, followed by visualization with a fluorescence-labeled secondary antibody for 1 hour at 37°C. DAPI was used to stain nuclei at room temperature. Images were captured and analyzed with ImageJ software.

**1.5 *In vitro* release assay of EVs from hydrogel**

To assess the release kinetic of EVs from GelMA hydrogel, PKH26 labeled EVs were incorporated into GelMA prepolymer to create EVs@GelMA hydrogel. The release assay was conducted using a transwell chamber system. hESCs were seeded in the lower chamber, while equal quantities of EVs solution (EVs in PBS) and EVs@GelMA hydrogel were introduced into the upper chamber. Fluorescence imaging and PKH26 fluorescence intensity measurements of the hESCs were performed at 24, 48, and 72 hours. The sustained protein release from EVs@GelMA was assessed using the bicinchoninic acid (BCA) method. In brief, EVs@GelMA at a concentration of 1 µg/µL were incubated in PBS at 37℃, with the supernatant collected every two days over an 18-day period. Protein concentration was quantified via a BCA assay kit, and the cumulative release of protein *in vitro* was plotted. All experiments were repeated 5 times, and results were averaged.

**1.6 Hematoxylin Eosin (HE), Masson and Immunohistochemical (IHC) Staining**

Mouse uterine tissues were embedded in paraffin and sectioned. HE staining was performed to observe morphological changes in the endometrium, including thickness and the number of glands. The fibrosis area ratio was detected by Masson staining and immunofluorescence stainning. For IHC staining**,** deparaffinized and dehydrated tissue sections were washed with PBS, incubated with 3% hydrogen peroxide for 10 minutes at room temperature, then conducted to PBS washing. After the addition of primary antibodies, incubated at 4℃ overnight. Afterward, the corresponding secondary antibodies were added, and DAPI was used to stain nuclei at room temperature. For HE/Masson staining, the tissue was fixed with 4% neutrally buffered formalin, followed by dehydration and embedding. The section thickness was 4 µm. Three high-power fields were selected for each HE/Masson-stained section. Subsequently, the number of glands, endometrial thickness and fibrosis area ratio were calculated individually, and the average values were then determined. Numerical analysis was conducted using ImageJ software. Fibrosis area ratio was calculated as follows: total area of endometrial fibrosis per field/the sum area of endometrium. The rate was automatically averaged using the Image-pro Plus software.

**1.7 Quantitative Real-Time PCR (RT-qPCR)**

Total RNA was extracted from cells using the RNeasy Mini Kit (QIAGEN, Germany). First-strand cDNA was generated with iScript™ cDNA Synthesis Kit (Bio-Rad, USA) and RT-qPCR assays

were carried out using the SYBR Green Premix kit (TaKaRa, Japan) according to the manufacturer's instructions. All corresponding primer information is displayed in Table S1.

**2. Supplementary Figures**


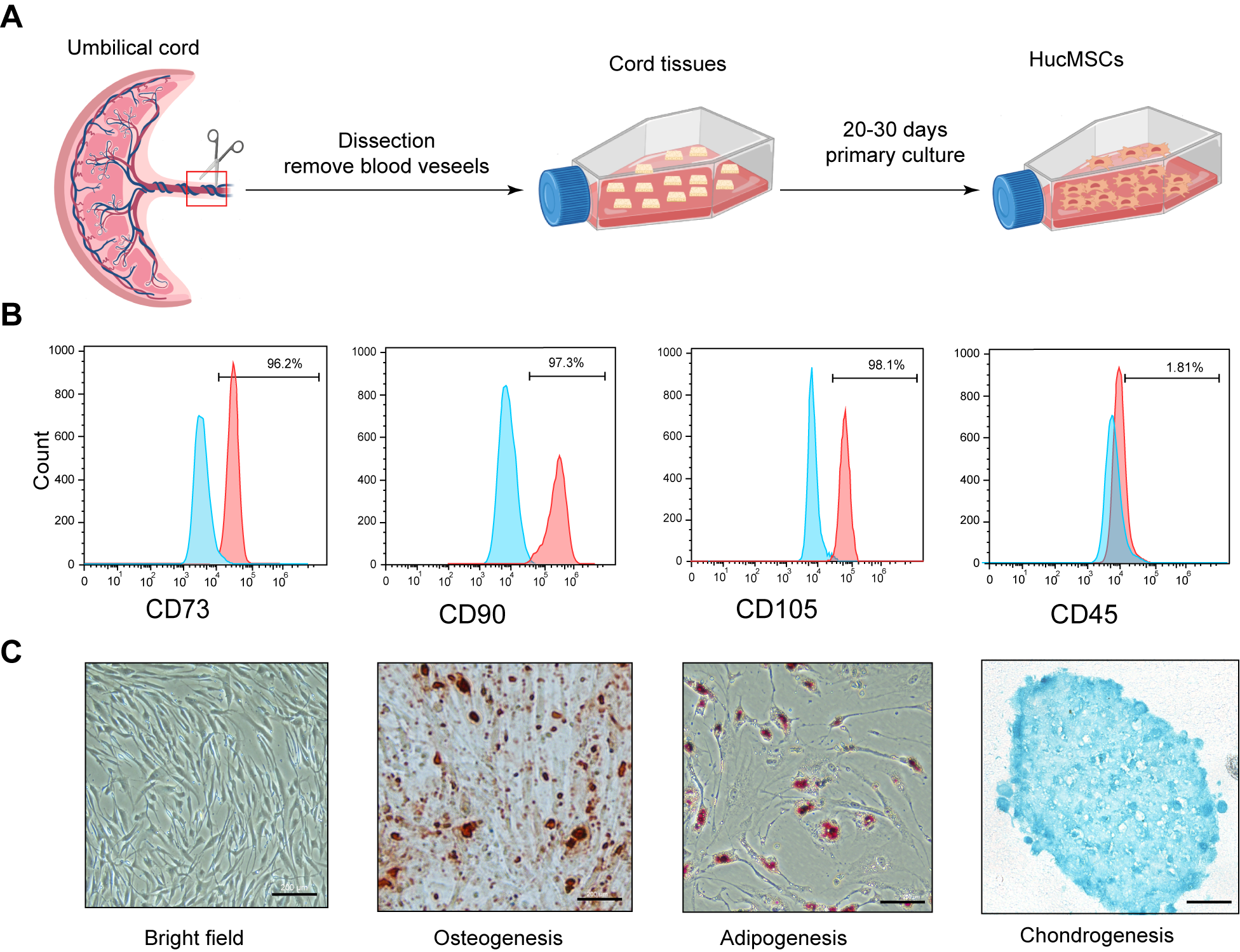


**Figure S1. Isolation and characterization of HucMSCs.** A. The workflow for HucMSC isolation. B. Flow cytometry analysis the expression of CD73, CD90, CD105, and CD45 of HucMSCs. C. Histological analysis of HucMSC differentiation ability into osteoblasts, adipocytes, and chondrocytes.


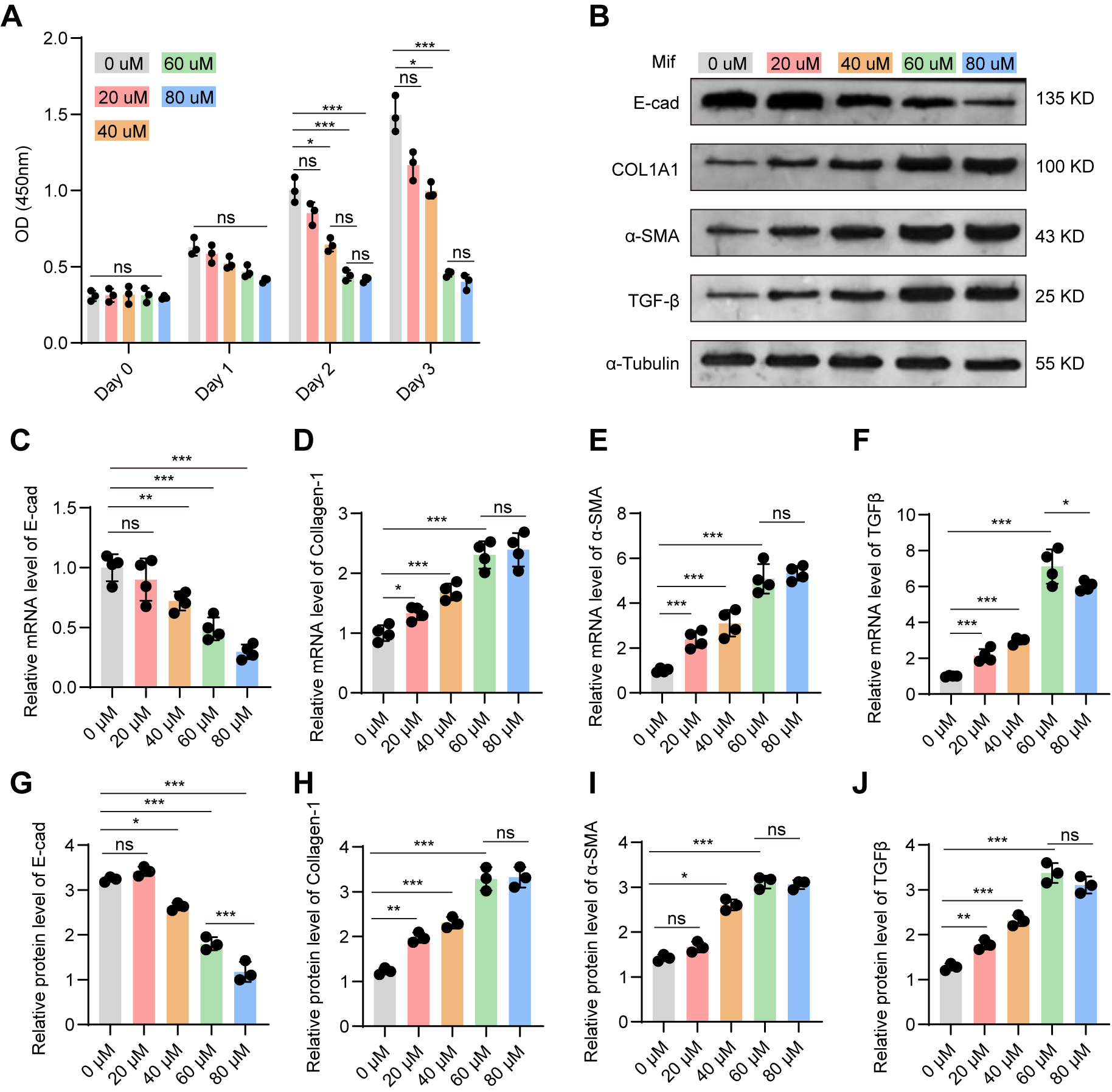


Figure S2. Validation of the optimal mifepristone concentration for establishing a hESCs injury model. (A) CCK-8 assay was performed to detect the viability of hESCs at different time points following incubation with 0, 20, 40, 60, and 80 μM Mif for 48 h, n=3. (B) Western blot analysis was performed to examine the dose-dependent effects of mifepristone on the protein expression levels of fibrosis markers (E-cadherin, Collagen-1, α-SAM, TNF-β) in hESCs, n=3. (C-F) RT-qPCR analysis was conducted to evaluate the dose-dependent effects of mifepristone on mRNA expression levels of the anti-fibrosis marker *E-cadherin* (C) and pro-fibrosis markers *Collagen-1* (D) , *α-SAM* (E), *TNF-β* in hESCs, n=4. G-J, Corresponding quantitative analysis of the Western blot results shown in (B). ns, not significant versus the indicated group. Data are presented as means ± SD.


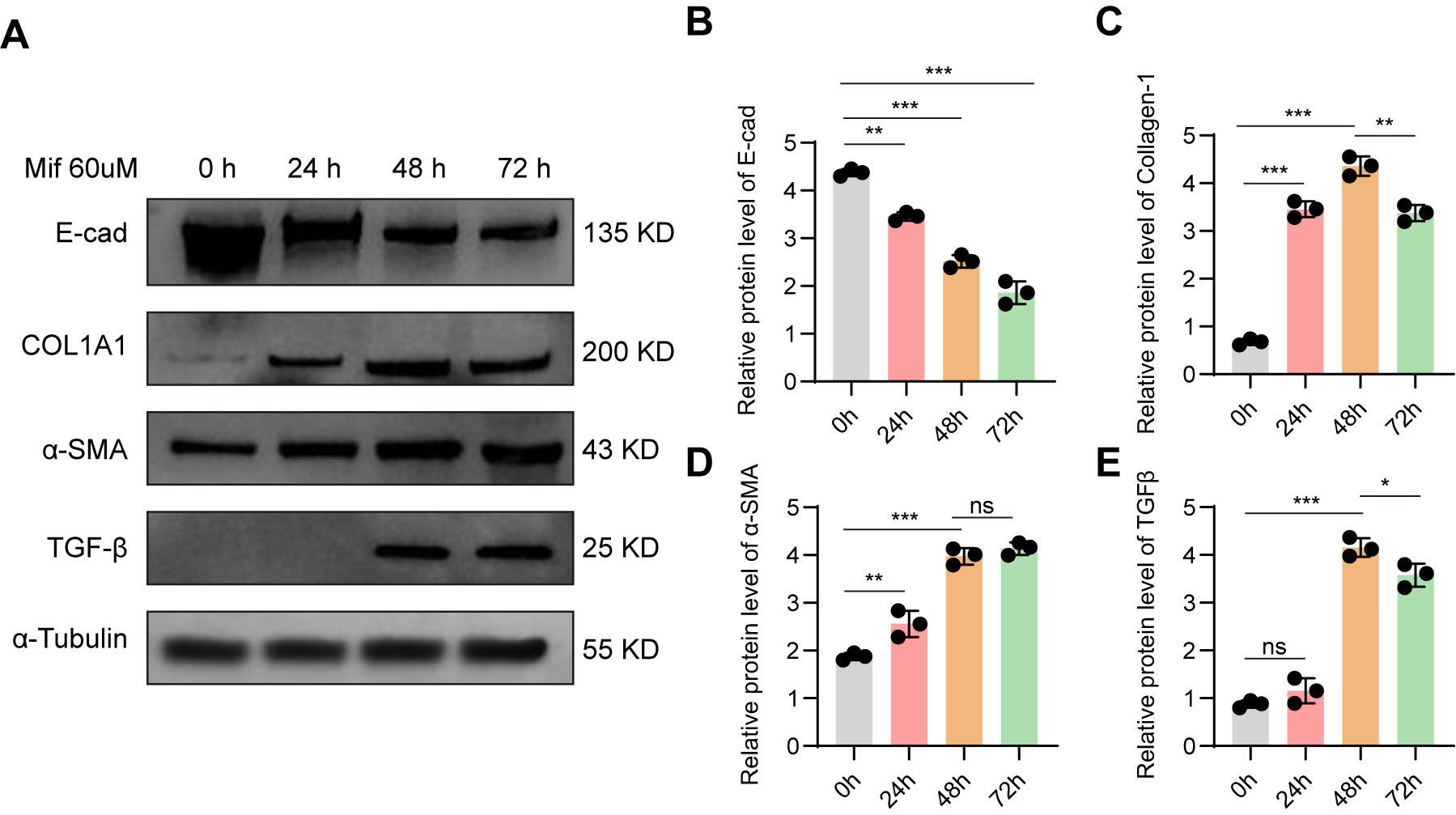

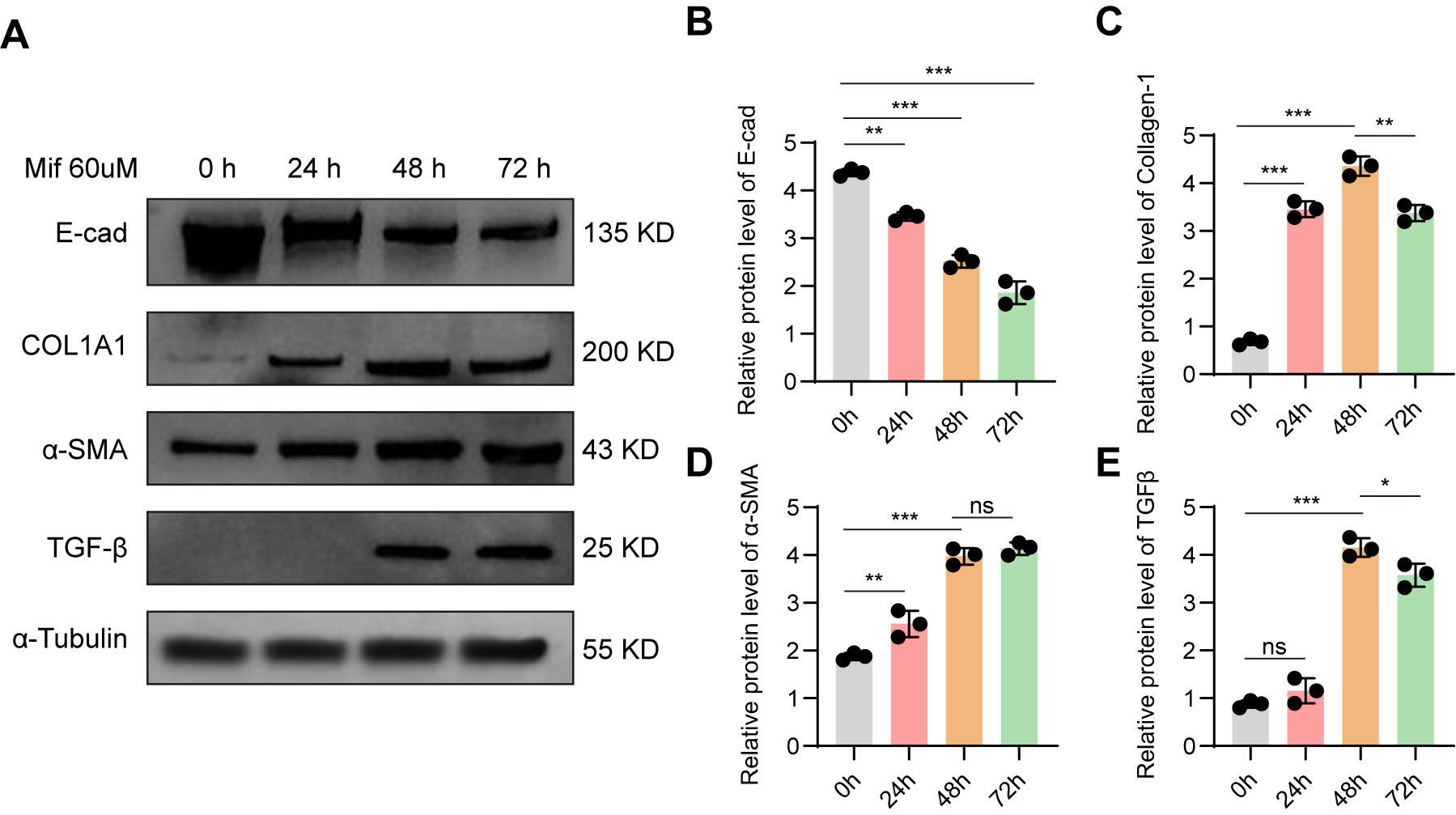


Figure S3. Optimization of mifepristone treatment duration for establishing an hESC injury model using 60 µM mifepristone. (A) Western blot analysis was performed to evaluate the time-dependent effects of mifepristone on the protein expression levels of fibrosis markers (E-cadherin, Collagen-1, α-SMA, TNF-β) in hESCs. (B-E) Corresponding quantitative analysis of the Western blot results shown in (A). ns, not significant versus the indicated group. ns, not significant versus the indicated group. Data are presented as means ± SD from n = 3 independent biological replicates.


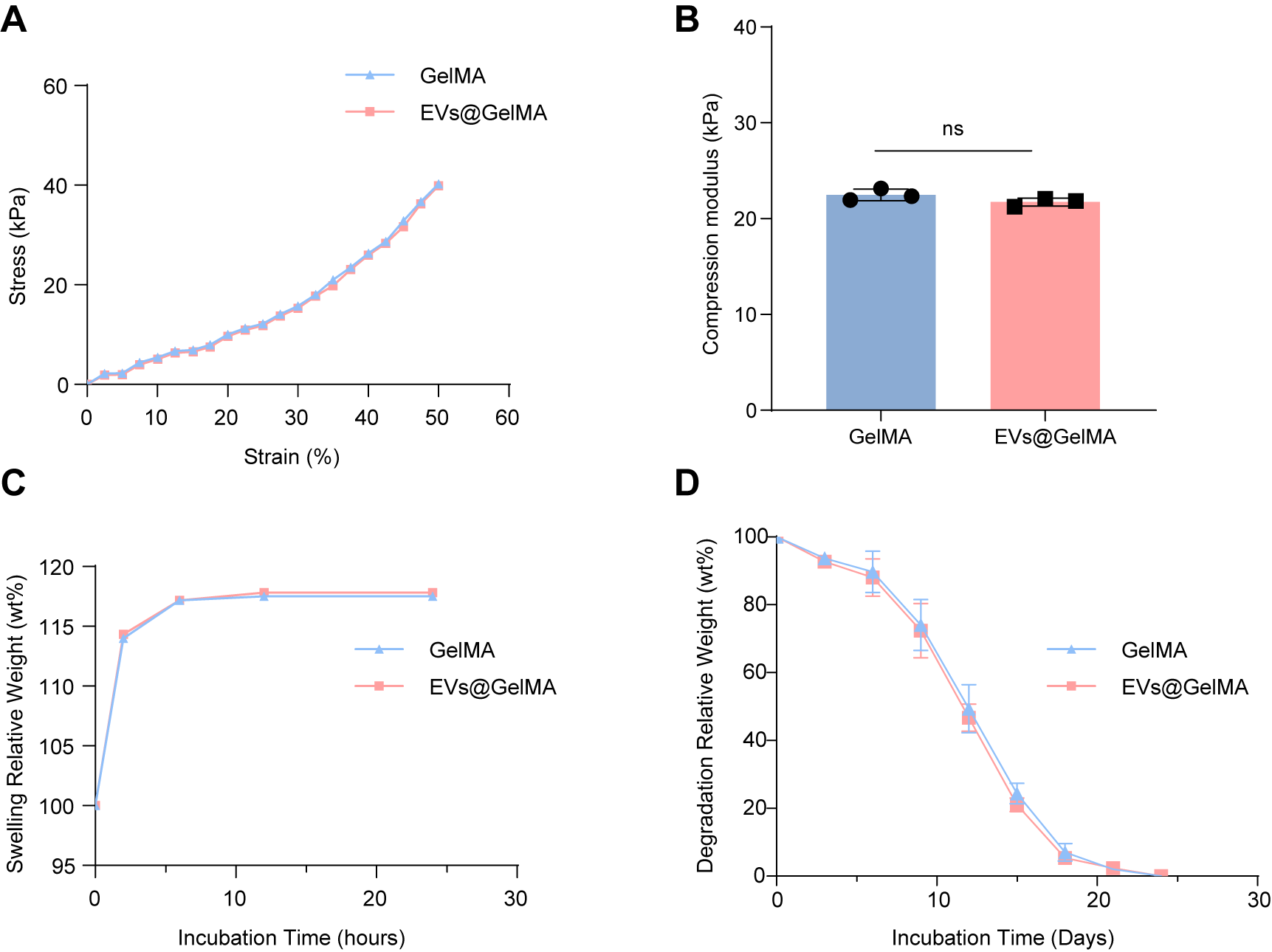


Figure S4. The mechanical property test data of the GelMA hydrogel. (A) Mechanical performance of 10% GelMA and EVs@GelMA. (B) Quantitative analysis the compression modulus of 10% GelMA and EVs@GelMA. (C) Swelling rate of 10% GelMA and EVs@GelMA. (D) Degradation rate of 10% GelMA and EVs@GelMA in vitro. ns, not significant versus the indicated group. Data are presented as means ± SD from n = 3 independent biological replicates.


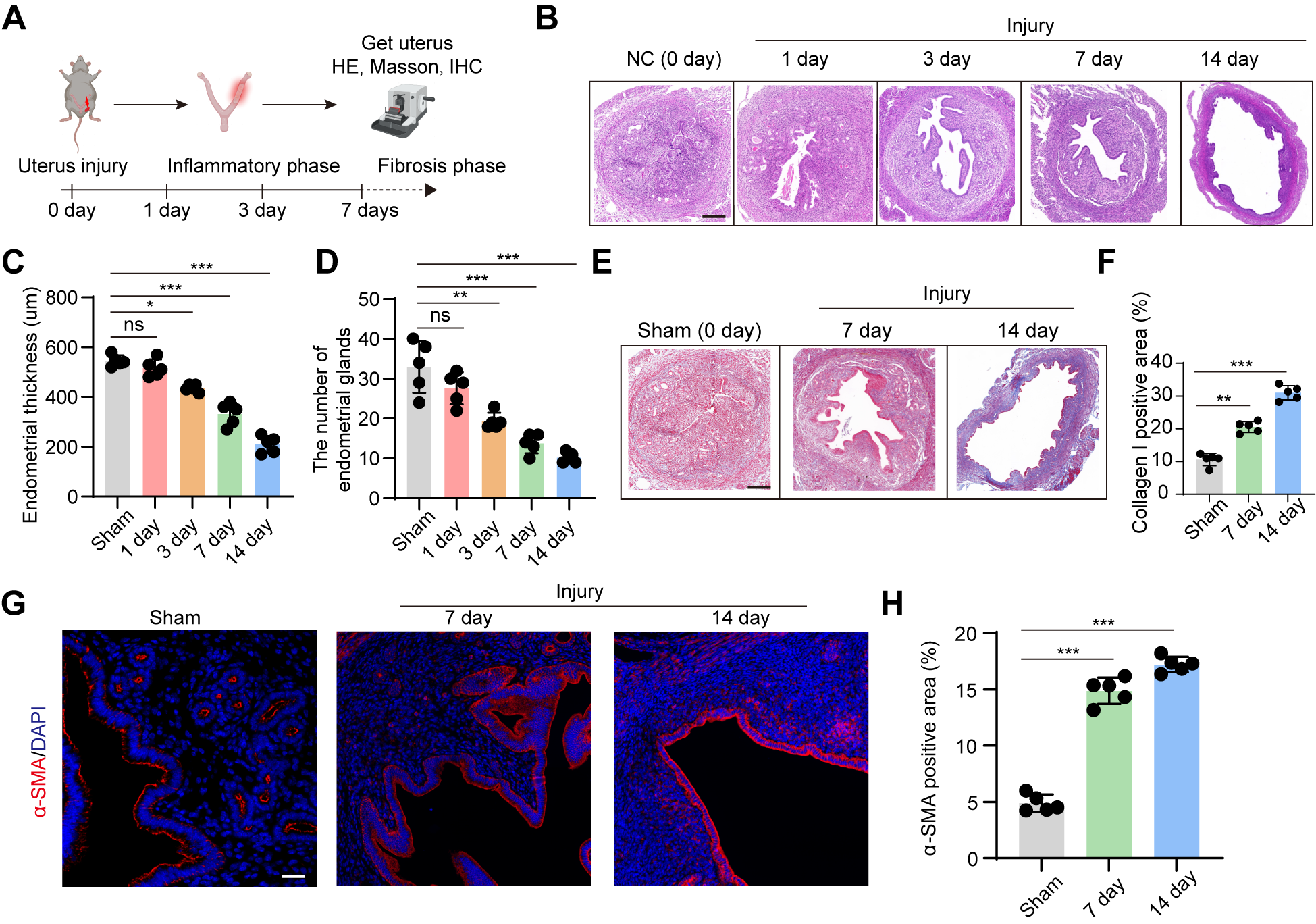


Fig S5. Pathological features of the IUA mice model. (A) The schematic diagram of modeling process. (B) HE staining of the endometrium in mouse uteri at 1, 3, 7, and 14 days after injury. (C) Statistical results of endometrial thickness corresponding to (B). (D) Statistical results of the number of uterine glands corresponding to (B). (E) Masson staining analysis of collagen deposition in the endometrium at different time points after uterine injury. (F) Statistical results of the Collagen Ⅰ-positive area corresponding to (E). (G) Immunohistochemical detection of α-SMA protein expression in the endometrium at different time points after uterine injury. (H) Statistical results of the α-SMA-positive area corresponding to (G). ns, not significant versus the indicated group. Data are presented as means ± SD from n = 5 independent biological replicates.


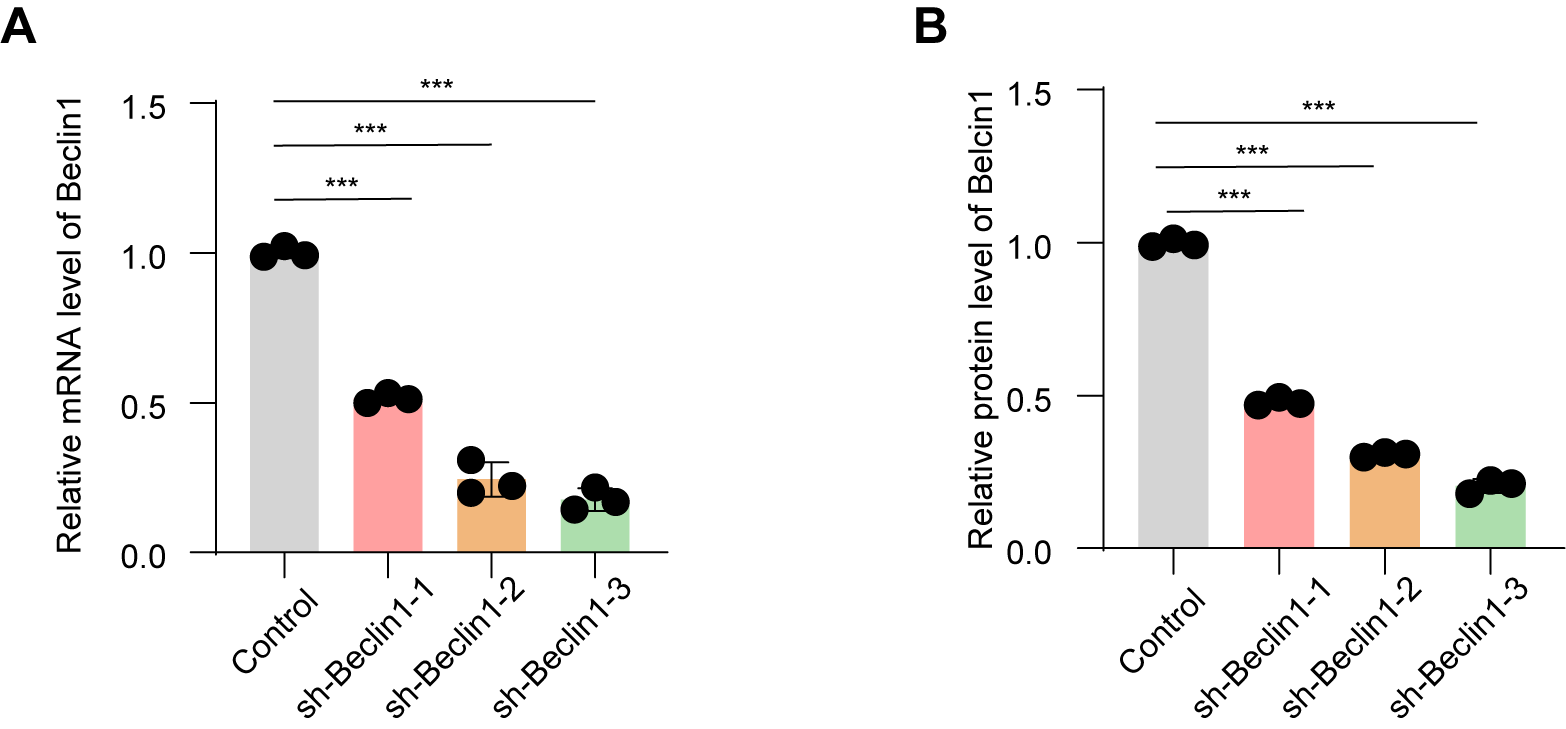


Figure S6. The knockdown efficiency of BECN1 was verified via RT-qPCR and Western blot analysis. (A) RT-qPCR detects *Beclin1* mRNA levels in hucMSCs after transfected with different shRNAs lentiviral. (B) Western blot analysis Beclin1 protein levels in hucMSCs after transfected different shRNAs lentiviral. Data are presented as means ± SD, with n = 3 independent biological replicates.


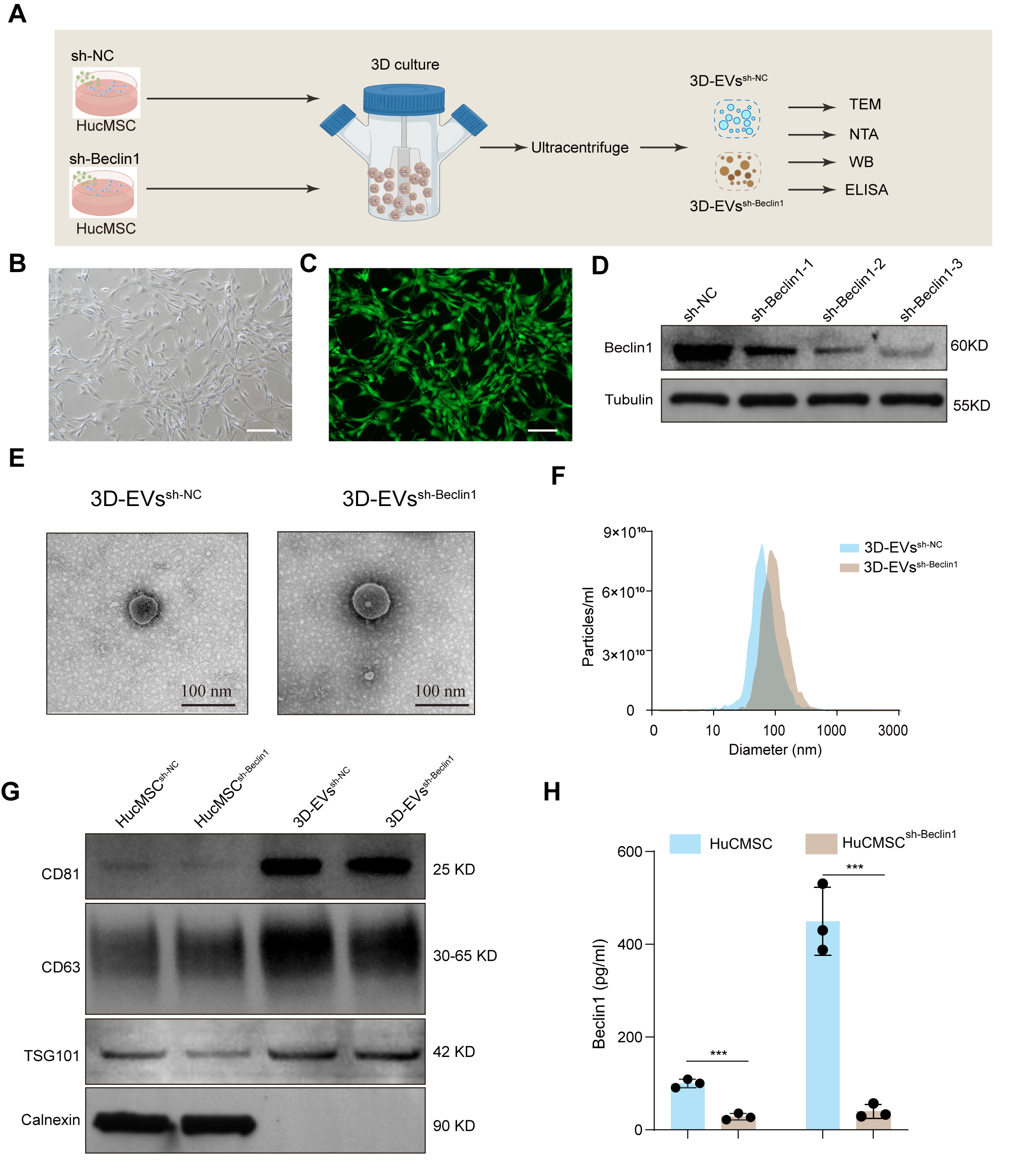


Figure S7. Knockdown of BECN1 in HucMSCs and their derived 3D-EVs. (A) Overview of the experimental workflow. (B-C) Morphological observation of HucMSCs following sh-BECN1 lentiviral infection. (D) Western blot analysis verifying the knockdown efficiency of BECN1 in HucMSCs. (E) TEM images of 3D-EVs derived from HucMSCs infected with sh-NC/sh-Beclin1 lentiviral. (F) Particle size distribution of 3D-EVs. (G) Western blot analysis of exosomal markers in HucMSCs and their secreted EVs. (H) Elisa analysis of BECN1 levels in HucMSCs and their secreted EVs. Data are presented as means ± SD, with n = 3 independent biological replicates.

**3. Supplementary Table**

**Table S1. Sequences of primers**

| **Gene** | **Forward** | **Reverse** |
| --- | --- | --- |
| E-cadherin | TCACCATTAACAGGAAACAGG | CCATCAGGCGTGACCCAGA |
| Collagen-1 | GAGGGCCAAGACGAAGACATC | CAGATCACGTCATCGCACAAC |
| α-SMA | CTATGCCTCTGGACGTACAACTG | CATCTCCAGAGTCCAGCACA |
| TGF-β | CGGAGAGCCCTGGATACCAA | CGAATCGTCTCCGACCTTGC |
| BECN1 | AAGACACAGTCCAGCCGGAT | GGCGTCTCAGCTGTCCGAGT |
| IGFBP5 | GAGACGCTGTGCGGAAGATG | CGTAGGTGTCTTGGTGAGGCT |
| GAPDH | GGACTGACCTGCCGTCTAG | TAGCCCAGGATGCCCTTGAG |

**Table S2. Sequences of shRNA**

| **Gene** | **Target sequence** |
| --- | --- |
| **shBeclin1-1** | GAGAGGAGCCATTTATTGA |
| **shBeclin1-2** | GCUUCUUGACUUGAAGAAAUU |
| **shBeclin1-3** | GGAGAAUGUACGAACUAUUUU |
